# The structuring of porous reticular materials for energy applications at industrial scales

**DOI:** 10.1039/d5cs00166h

**Published:** 2025-04-08

**Authors:** Mehrdad Asgari, Pablo Albacete, Dhruv Menon, Yuexi Lyu, Xu Chen, David Fairen-Jimenez

**Affiliations:** a The Adsorption and Advanced Materials Laboratory (A^2^ML), Department of Chemical Engineering and Biotechnology, University of Cambridge Philippa Fawcett Drive Cambridge CB3 0AS UK ma2000@cam.ac.uk df334@cam.ac.uk; b Departamento de Química Inorgánica, Universidad Autónoma de Madrid 28049 Madrid Spain

## Abstract

Reticular synthesis constructs crystalline architectures by linking molecular building blocks with robust bonds. This process gave rise to reticular chemistry and permanently porous solids. Such precise control over pore shape, size and surface chemistry makes reticular materials versatile for gas storage, separation, catalysis, sensing, and healthcare applications. Despite their potential, the transition from laboratory to industrial applications remains largely limited. Among various factors contributing to this translational gap, the challenges associated with their formulation through structuring and densification for industrial compatibility are significant yet underexplored areas. Here, we focus on the shaping strategies for porous reticular materials, particularly metal–organic frameworks (MOFs) and covalent organic frameworks (COFs), to facilitate their industrial application. We explore techniques that preserve functionality and ensure durability under rigorous industrial conditions. The discussion highlights various configurations – granules, monoliths, pellets, thin films, gels, foams, and glasses – structured to maintain the materials’ intrinsic microscopic properties at a macroscopic level. We examine the foundational theory and principles behind these shapes and structures, employing both *in situ* and post-synthetic methods. Through case studies, we demonstrate the performance of these materials in real-world settings, offering a structuring blueprint to inform the selection of techniques and shapes for diverse applications. Ultimately, we argue that advancing structuring strategies for porous reticular materials is key to closing the gap between laboratory research and industrial utilization.

## Abstract 2

Advancing structuring strategies for porous reticular materials is key to closing the gap between laboratory research and industrial utilization.

## Graphical abstract

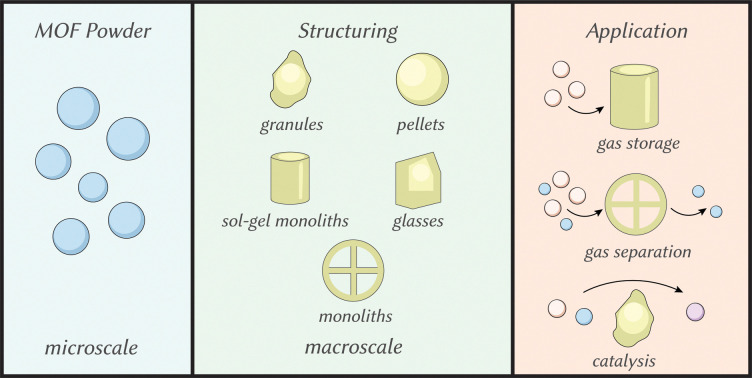

## 1. Introduction

**Fig. 1:**
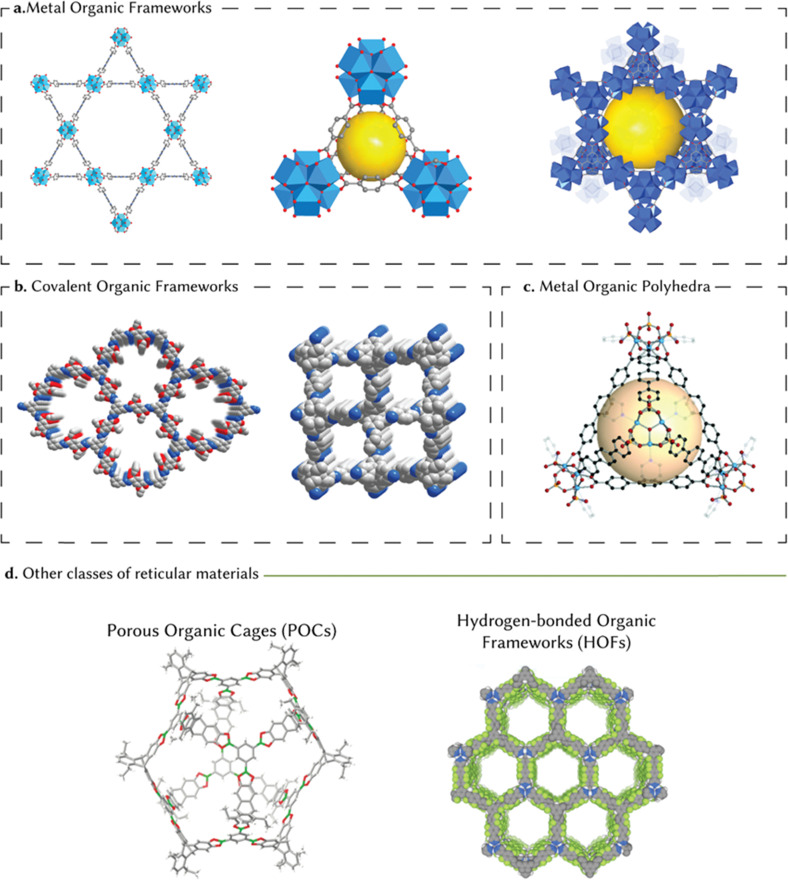
An introduction to porous reticular materials. (a) MOFs or PCPs are metal ions or metal-containing nodes connected by organic linker molecules using coordination bonds. Due to the large length of the linker molecules, MOFs show a high degree of porosity. Here, we present crystal structures of archetypal MOFs with high porosities: PCN-222, UiO-66, and MOF-808. (b) COFs are covalent porous crystalline polymers formed by the integration of organic building blocks into ordered structures. These structures are typically lightweight and tend to have low mass densities. Here, we present crystal structures of archetypal COFs: COF-42 and COF-300. (c) MOPs are coordination cage compounds formed through linking metal cations with organic linkers. As opposed to MOFs, these cages exist in isolation – however, they can serve as building blocks for creating extended solids. (d) Outside of these classes of reticular materials, in recent years, there has been developments into new families such as POCs and HOFs. Figure has been adapted with permission from ref. 11 Copyright 2015 American Chemical Society, ref. 12 Copyright 2015 Oxford Academic, ref. 10 Copyright 2021 Royal Society of Chemistry, and ref. 13 Copyright Elsevier 2022.

Establishing precise control over the size and uniformity of the porous space has multiple implications for large-scale applications. Materials that exhibit a high degree of porosity are of interest across diverse technologies due to their ability to interact with other chemical species, such as gases and liquids, not only at their external surface but also throughout their internal porosity.^1^ Beyond classical porous materials such as activated carbons and zeolites, the synthesis of permanently porous structures in reticular materials or coordination polymers was, until the late 1990s, thought to be largely unfeasible based on the age-old perception that ‘nature abhors vacuum’.^2^ The emergence of the ‘reticular chemistry’ concept facilitated the geometry-guided design of periodically extended, crystalline structures by linking molecular building blocks through strong bonds – leading to the creation of new porous materials. These rigid building blocks assemble into predetermined target networks, retaining their structural integrity and rigidity throughout the synthesis process.^3–5^ The presence of strong bonds facilitates the generation of crystalline frameworks with high architectural stability – overcoming a critical challenge that previously held back the realization of permanently porous solids.^6^ This pioneering class of reticular materials were referred to as metal–organic frameworks (MOFs)^7^ or porous coordination polymers (PCPs),^8^ but also expanded into sister families such as covalent organic frameworks (COFs)^9^ and metal–organic polyhedra (MOPs)^10^ – summarized in Fig. 1 – as well as porous organic cages (POCs) and hydrogen-bonded organic frameworks (HOFs).

MOFs and PCPs are constructed by connecting metal ions or metal-containing nodes with organic linker molecules using coordination bonds (Fig. 1a). Due to the relatively strong bonds and potentially large length of the linker molecules, MOFs can show a high degree of porosity (up to 90% of void volume), and large specific surface areas (1000–10 000 m^2^ g^−1^).^7^ In addition, the high flexibility in terms of the choice of precursors facilitates the realization of virtually infinite possible structures, each tailored to the application at hand. So far, more than 100 000 PCPs/MOFs have been reported in the literature, and about 15% of them are porous.^7,14^ Similar principles of topology-guided design have led to the synthesis of COFs, covalent-bonded, porous crystalline polymers that integrated organic building blocks into ordered structures (Fig. 1b).^9^ The use of covalent bonds to connect molecular building blocks has, historically, led to the construction of amorphous or poorly defined materials. However, in the case of COFs, this problem was overcome by the use of B–O, C–N, B–N, and B–O–Si linkages.^11^ In addition, MOPs are coordination cage compounds formed by the linkage of metal cations and organic linkers that, in contrast to MOFs, exist as isolated cages. MOPs can also be used as secondary building blocks to create extended solids, such as MOFs (Fig. 1c).^10,15^ Like MOPs, POCs are discrete, cage-like structures created through the covalent bonding of organic molecules, without metals. These structures are notable for their solution processability and adjustable pore sizes. On the other hand, HOFs represent a class of porous materials formed by hydrogen bonding between organic molecules. Unlike the coordination bonds in MOFs or the covalent bonds in COFs, hydrogen bonds in HOFs provide a balance between structural rigidity and flexibility. This characteristic makes HOFs potentially valuable for applications requiring reversible assembly and disassembly, such as in molecular storage, and selective separation processes.^13^

**Fig. 2:**
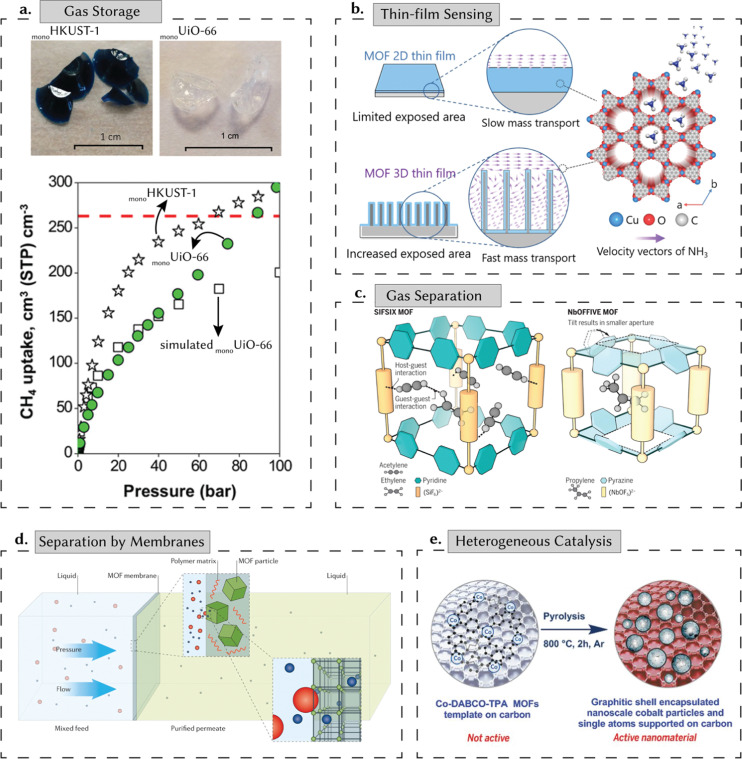
Energy applications of porous reticular materials. (a) Gas storage: high surface areas and the prospect of incorporating multiple functionalities make reticular materials ideal for gas storage. In recent years, monolithic MOFs have made tremendous strides on this front, surpassing the target set by the US Department of Energy (dashed red line) for methane (CH_4_) storage. (b) Thin-film sensing: porous reticular materials have been used in sensing, electronics, and optics, enabling precise control and manipulation of light, electrons, and chemical species. As an illustrative example, we present a 3D-MOF thin film with good crystallinity and precisely controllable thickness with high sensitivity and fast response for ammonia (NH_3_) sensing. Adapted with permission from ref. 19 Copyright 2021 Wiley. (c) Gas separation: the porous nature of reticular materials makes them effective for complex gas separations. Here, we show the design of two MOFs – each specialized for a specific gas separation. SIFSIX effectively sieves acetylene over ethylene, while NbOFFIVE selectively sieves propylene over propane. Adapted with permission from ref. 20 Copyright 2016 American Association for the Advancement of Science. (d) Separation by membranes: filtration separation across a MOF-based membrane. These separations typically rely on the pore size or the rate of diffusion. Species smaller than the pore diameter permeate the membrane, while larger species are unable to permeate – leading to a selective separation. Adapted with permission from ref. 21 Copyright 2016 Springer Nature. (e) Heterogeneous catalysis: high surface areas allow high reaction rates per unit volume, and the tailorability of the porous structure allows rapid transport of reactant and product molecules. Here, we present the design of a MOF-based cobalt nanoparticle that catalyses the synthesis of amines. Adapted with permission from ref. 22 Copyright 2017 American Association for the Advancement of Science.

While these new porous materials have potential for multiple applications, the underlying principles surrounding their selection are centered around their adsorption properties, In particular, adsorption properties for either the storage, separation (or capture), and release of chemical entities such as gases, ions, or liquids within their void space. Fig. 2 shows key, potential, energy-related applications for reticular materials that are covered in this review: (i) gas storage, (ii) gas separation, (iii) separation by membranes, (iv) heterogeneous catalysis, and (v) thin-film-based sensing. Gas storage is crucial in various industries, primarily concerning energy stability and alternative fuel sources.^16^ Nowadays, the most important gaseous energy vector of interest is, arguably, hydrogen. It is a key element in the necessary, green energy transition and de-carbonization.^17^ Here, the high surface area and pore volume of porous materials are important, as they are strongly correlated to their gravimetric adsorption capacity. However, the material's density is another – very often ignored – parameter, as together with the pore volume, it defines the volumetric adsorption capacity: this is, the amount of gas one can store in a fixed amount of volume.^16^ Indeed, most industrial applications, including hydrogen storage and carbon capture, have limitations on the space that can be used to be implemented. Recent advancements in shaped, densified, MOFs (Fig. 2a) – called monoliths due to their single-form factor – have surpassed methane storage targets set by the US Department of Energy, highlighting their potential to meet evolving energy demands while accommodating diverse functional requirements.^18^

For thin films, porous reticular materials have been proposed for sensing, electronics, and optics, enabling precise control and manipulation of light, electrons, and chemical species – impacting environmental monitoring, safety, and automation.^23^ They have transformed these fields, as seen in 3D-MOF thin films (Fig. 2b), exhibiting high sensitivity and rapid response in ammonia sensing. While we discuss these applications in some detail in the sections that follow, for better context, we refer readers to dedicated reviews on these topics.^17,23–26^ Porous reticular materials have also been proposed for gas separations such as carbon dioxide, carbon monoxide, ammonia, and hydrocarbons. Their porosity can be tuned to facilitate molecular sieving effects or promote specific interactions for selective adsorption.^24^ For example, MOFs such as SIFSIX and NbOFIVE, constructed from fluorinated clusters and pyrazine/pyridine-based ligands, show their efficacy in hydrocarbon separation and carbon capture (Fig. 2c).^20^ Their adjustable porosity enables precise molecular sieving effects and selective adsorption tailored to different gases. Moreover, the separation properties of reticular materials can also be incorporated into membranes, enabling molecular sieving of gases, ions (*e.g.*, toxic, heavy metals), and liquids based on differences in size, shape, and chemical affinity.^24,25^Fig. 2d shows a schematic diagram of MOF-based membranes incorporated into a polymer matrix, suitable for various applications including water purification. For heterogeneous catalysis, reticular materials can outperform conventional materials in certain reactions. Here, their potential is based on their high surface areas that allow high reaction rates, the presence of functional groups and metals to promote the reactions, and the tailorability of the porous structure to allow for rapid transport of reactant and product molecules while offering the possibility of shape-selective catalysis.^26^ Techniques such as pyrolysis of MOF-based materials can enhance catalytic activity further by generating a porous carbon matrix containing single active catalytic sites (Fig. 2e).^22^

**Fig. 3:**
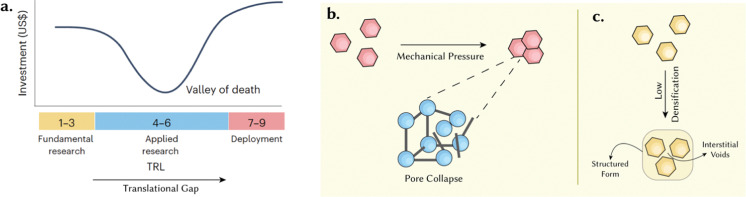
Translational gap towards the commercialisation of porous reticular materials. (a) Typical investments required at each TRL – with the slump at TRLs 4–6 corresponding to the translational gap. Adapted with permission from ref. 27 Copyright 2024 Springer Nature. Structuring as a crucial aspect of the translational gap. (b) A poorly chosen structuring technique – for instance mechanical pressure – may cause a collapse of the porosity. (c) Low densification may lead to interstitial voids.

Despite the excellent performance metrics and promise of reticular materials, their successful translation to industry is limited. Following 25 years since their inception and very few notable exceptions,^27^ their study is still largely confined to laboratory-based studies.^28^ Indeed, the commercialization of materials technologies spans long timescales – with reports suggesting approximately 5–15 years to transition from discovery to the commercial market.^29^ Technology Readiness Levels (TRLs) provide a strong framework for appreciating the associated costs and timescales for commercialization.^27^ TRLs of 1–3 correspond to fundamental research, 4–6 imply applied research – focusing on prototyping and development, while TRLs of 7–9 focus on commercial-scale deployment (Fig. 3a).^27^ Here, arguably, TRL 4–6 is the most challenging phase – often termed the ‘valley of death’ – as it lies between stages where public funding is limited while private capital is difficult to secure. More advanced MOF-based technologies – such as Svante's development of CALF-20 laminates^30^ and Immaterial's sol–gel, monolithic solutions^31^ for CO_2_ capture from wet acidic gas streams – lie in this phase.^27,32^ As such, we term the region between TRLs 1–3 and TRLs 7–9 as the ‘translational gap’. Several aspects are responsible for this translational gap, including cost, hydrochemical, and mechanical stability. While cost is a function of raw materials, process synthesis and scale, stability is still one of the most important concerns for long-term operability – particularly in applications subject to harsh industrial conditions such as carbon capture.^33^ Crystalline porous materials are considered metastable with respect to their dense phase.^33^ In the case of MOFs, this refers to dense, amorphous phases obtained either through the thermal route^34^ or *via* mechanical pressure.^34,35^ In this regard, the structuring and densification is an issue that – as stated above – has been relatively ignored in the academic literature but is critical in industrial applications. Owing to this metastability, structuring is a crucial aspect because poorly chosen structuring techniques using, *e.g.*, mechanical pressure, often lead to a partial collapse in the material's porosity (Fig. 3b). On the other hand, poor structuring and densification can also lead to low densities due to the existence of interstitial space between the material particles – a volume that, generally, cannot be exploited in adsorption, increasing the footprint of designed systems, and reducing the promised volumetric performance of MOFs (Fig. 3c). Both options – pore collapse and low densification – are detrimental to their performance, ultimately defeating the purpose of employing porous materials (Fig. 3b and c).

The structuring and densification of reticular materials have broad implications, for their industrial application. In this review, we use the terms ‘structuring’ and ‘shaping’ interchangeably, as these processes are inherently correlated in the present context. While ‘shaping’ typically refers to the macroscopic transformation of materials into functional forms, ‘structuring’ focuses on maintaining and optimizing the internal architecture, including pore size, connectivity, and hierarchical organization. Unlike conventional materials, where shaping may compromise structural integrity, reticular materials must be shaped while preserving microstructural features to retain their properties at macro-scales. Traditional shaping techniques often risk pore collapse, reduced surface area, or loss of crystallinity, making it essential to incorporate structuring principles to maintain performance. Advanced processing techniques such as gel casting, additive manufacturing, and templated assembly simultaneously shape and structure these materials, ensuring their functional properties are retained. Given the close relationship between these concepts, and the fact that porous materials research often overlaps these definitions, we have adopted a flexible approach in using these terms throughout this review.

In this review, we explore various strategies for structuring porous reticular materials, including MOFs, COFs, HOFs, and other emerging frameworks, focusing on their implications for energy applications. It is important to acknowledge, however, that MOFs have received significantly more attention in both academic and industrial contexts. This is due to their earlier discovery, structural diversity, and well-established synthetic protocols, which have enabled their widespread adoption across applications of interest. Compared to other classes of reticular materials, MOFs have undergone extensive optimization and scale-up efforts, being, currently, the most advanced candidates for industrial implementation.^27^ Consequently, many structuring techniques have been developed and refined specifically for MOFs, leading to their predominant focus in this review. However, we also incorporate key examples of COF structuring strategies to highlight the broader relevance of these methods across different porous frameworks. Our discussion aims to provide a strong understanding of structuring challenges and opportunities that are in principle, applicable across reticular materials, while placing a bulk of the emphasis on MOFs due to their closer proximity to large-scale applications.

We focus here on energy applications, but the underlying principles are universal, and the described techniques are transferable to applications and materials in different contexts. Section 2 focuses on the hierarchical synthesis of porous, reticular materials at the microscale and their size- and shape-controlled synthesis, templated approaches, and self-assembly of superstructures. Section 3 describes the landscape of available shapes for structuring porous reticular materials. Section 4 describes strategies for achieving these shapes at the macroscale and how these strategies would impact the material's performance. Section 5 discusses key considerations for the industrial translation. Here, we distinguished between the shape and the shaping technique because each form may be achieved using different techniques. At the end of the day, the choice of technique will depend on the scale of production, the associated costs, and the desired quality of the material at the macroscale. Considering the vast application landscape of these materials, rather than providing comprehensive discussions about specific applications, the goal is to provide a holistic, structuring ‘blueprint’ for bridging the translational gap.

## 2. The hierarchical synthesis of reticular materials at the microscale

A key feature of reticular materials is their metastable crystalline structure with empty pores. For industrial applications, one needs to ensure that the energy barrier is high enough for the transition from a porous crystalline to an amorphous phase to avoid pore collapse,^33^ or that there are alternative methods for shaping and densification that avoid this issue. In the first case, this requires the materials to survive high mechanical pressures, which necessitates control over the micro and sub-microscale architecture of reticular materials.^36^ Given that these structures arise from coordination-driven molecular self-assembly,^37^ the main challenge involves directing such reactions to create superstructures.^38^ Advances in synthetic and fabrication technologies allow for significant control at these scales. Here, we introduce these techniques, setting the stage for discussing the structuring of reticular materials at the macroscopic level without covering the basic theory behind material formation. For in-depth discussions, we refer readers to specialized reviews on this topic.^39,40^

### 2.1. Size-controlled synthesis

**Fig. 4:**
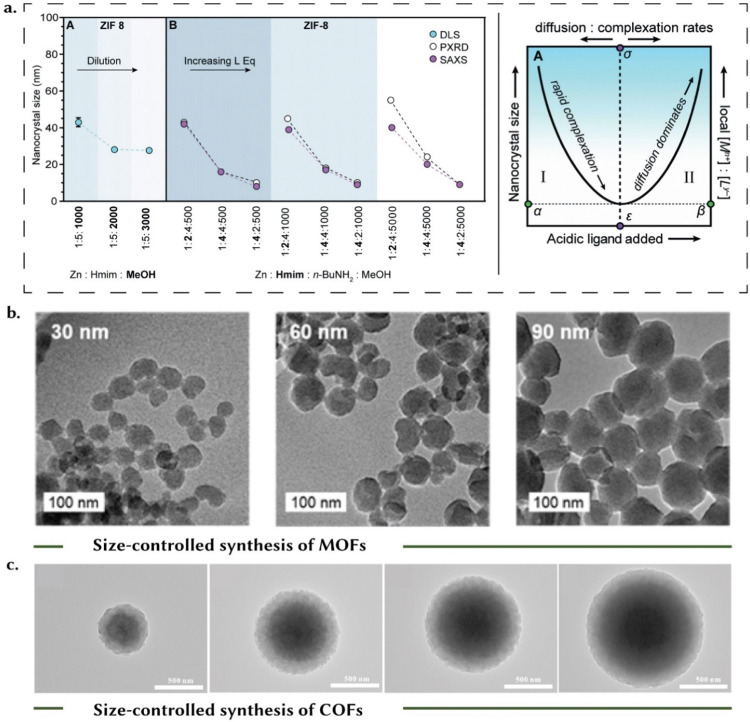
Size-controlled synthesis of reticular materials. (a) Left: Trends in the crystal sizes of ZIF-8 upon varying concentrations of the metal (Zn), linker (1-methylimidazolium, Hmim), and the modulator (*n*-butylamine, *n*-BuNH_2_). Excess concentrations of the linker deplete the local concentration of metal ions, thereby causing smaller crystal sizes even in the absence of a modulator. Right: There exists a ‘seesaw’ relationship between the crystal size and the concentration of acidic linker/modulator. At concentrations below the minima, increasing the ligand concentration leads to a decrease in particle size due to the depletion of local metal concentrations. At concentrations above the minima, increasing the ligand concentration interferes with the deprotonation mechanism. Adapted (modified) with permission form ref. 43 Copyright 2019 Royal Society of Chemistry. (b) Size control of MOFs – as an illustrative example, we present the downsizing of PCN-224 from a millimeter scale to the nanoscale which was achieved by diluting the system. This approach preserves the phase purity of the system, as it does not change the stoichiometry, while facilitating the creation of more MOF monomers – resulting in smaller particle sizes. Adapted (modified) with permission from ref. 47 Copyright 2016 American Chemical Society. (c) Size control of COFs: manipulating the concentration of a modulator facilitated size-control of a spherical COF. Adapted with permission from ref. 48 Copyright 2019 American Chemical Society.

In industrial applications, the performance of the macroscale, shaped material depends on the particle size – or rather the dispersity of the particle size – at the microscale. Good control over the dispersity could help reduce the interstitial space formed upon densification through a particle size distribution approach.^41,42^ However, while these processes are conceptually well-defined, conventional models may struggle to fully capture the complexities of nucleation and growth phenomena, especially in the case of MOFs and similar materials.^43^ Take, for example, the LaMer model,^40,44^ which distinguishes nucleation and growth, attributing distinct thermodynamic driving forces to each due to high precursor concentrations. While this model aligns with observations for certain MOFs, such as MOF-5,^45^ where crystal nucleation and growth distinctly occur, for other MOFs, such as HKUST-1,^46^ these phases appear to overlap, suggesting a simultaneous occurrence. Given these observations, Brozek *et al.*^43^ show that the kinetic control of chemical parameters to arrest particle growth is necessary for the modulation of particle size. The kinetic entrapment of MOF crystal size regimes relies on the interplay of competitive chemical equilibria, which encompass: (i) linker deprotonation, (ii) modulator deprotonation, (iii) linker complexation, and (iv) termination.^43^ To form a metal-linker bond, typically, the linker must undergo deprotonation, and a similar deprotonation process is required for the modulator to function effectively. Furthermore, both the linker and the modulator compete for coordination sites with metal ions, and so, control over these two reactions allows regulation of particle size. The degree of complexation between the metal and the linker also plays a pivotal role in governing growth. The effective arrest of particle growth occurs when the concentration of the linker significantly surpasses that of the metal ion. This phenomenon arises from the fact that higher equivalent linker concentrations enhance metal-linker complexation, consequently depleting available metal-ion concentrations. In essence, higher concentrations of linkers or modulators tend to typically result in smaller nanocrystal sizes. In each of the four equilibria discussed above, the prevalence of fast-forward reaction rates and limited reversibility tends to favor the formation of bulk structures. Modulators, compounds that can control the crystallization process are also important. Fig. 4a illustrates the impact of these parameters, showing the variation in crystal sizes of ZIF-8 across different concentrations of zinc metal, linker (1-methylimidazolium, Hmim), modulator (*n*-butylamine, *n*-BuNH_2_).^43^ Notably, excessive linker concentrations alone can diminish local metal ion availability, resulting in smaller crystal sizes even without a modulator present. This highlights the critical role of precursor ratios in finely modulating crystal growth dynamics in MOFs. Fig. 4a (right), shows a ‘seesaw’ relationship between the acidic linker or modulator concentrations and crystal size. Below a specific concentration threshold, increasing the ligand concentration reduces particle size due to metal ion depletion, showing the sensitivity of crystal growth to precursor ratios and local chemical environments. Conversely, surpassing this threshold can disrupt the deprotonation mechanism essential for crystal growth, leading to larger particle sizes. This dual effect emphasizes the rather delicate balance required in precursor concentrations to achieve desired crystal sizes in frameworks like ZIF-8. Such size control strategies could be applied to other porous reticular materials as well. For instance, the downsizing of PCN-224 from millimeter to nanoscale (Fig. 4b) dimensions through system dilution preserves phase purity and stoichiometry while promoting the formation of smaller MOF monomers.^47^ Expanding to COF synthesis, the manipulation of modulator concentration is shown to facilitate size control of a spherical COF (Fig. 4c), thus representing another approach to tailor the physical characteristics of porous materials, offering insights into how chemical manipulation can influence their structural properties.^48^ In some cases, modulators work by altering the pH, thereby influencing the kinetics of linker deprotonation. Higher pH values accelerate linker deprotonation, leading to the formation of smaller particles. On the other hand, lower pH values slow down deprotonation, causing nucleation to occur over extended periods, and ultimately yielding larger crystals. In other cases, modulators act as coordination agents, competing with the linker during complexation.^49^

### 2.2. Shape and morphology-controlled synthesis

**Fig. 5:**
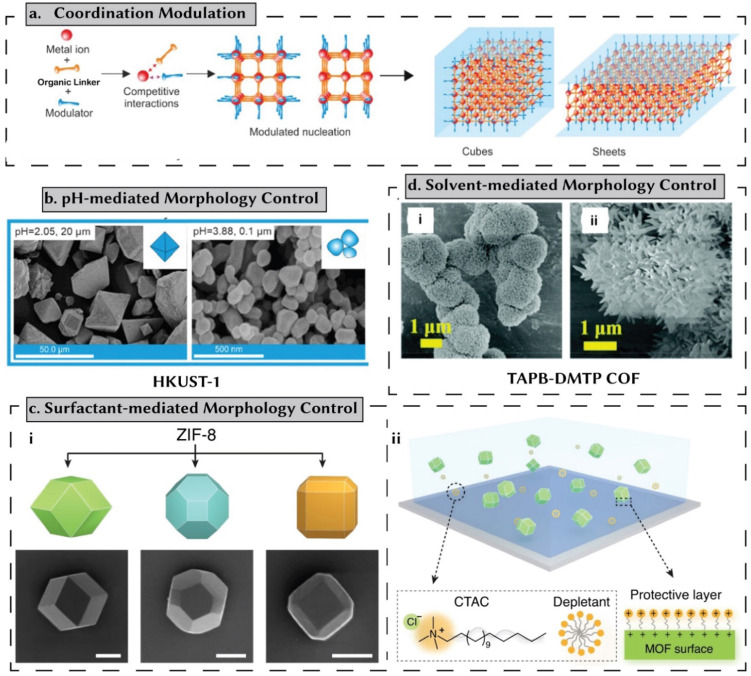
Shape-/morphology-controlled synthesis of reticular materials. (a) In a coordination modulation approach, the modulator and the linker compete for metal-ions. A strong control over these competitive interactions facilitates morphology control. Reprinted with permission from ref. 50 Copyright 2023 Elsevier. (b) Higher pH levels tend to shift the linker deprotonation equilibria toward the forward direction, accelerating nucleation rates and consequently affecting morphology. This approach is demonstrated here, for HKUST-1. Reprinted with permission from ref. 50 Copyright 2023 Elsevier. Data originally reported in ref. 54. (c) Surfactants exhibit a preference for attaching to specific crystal facets due to favorable interaction energies. Consequently, certain facets experience faster growth rates compared to others, primarily because these molecules either facilitate or hinder the attachment of reactant species to these facets. Reprinted with permission from ref. 55 Copyright 2023 Springer Nature. (d) Morphology control of COFs achieved by adjusting the solvent and inhibitor ratio: H_2_O/PEG-400; (i) 0.5 mL/0.5 mL and (ii) 0.5 mL/1 mL. Reprinted with permission from ref. 56 Copyright 2022 Royal Society of Chemistry.

In addition to size control, modulators and templates modify the nucleation and growth mechanisms in porous materials, allowing the ability to control their shape and overall morphology.^50^ Similar to size control, the main parameters that control shape are:

(i) Coordination modulation: this method involves competition between the modulator and the linker for metal ions. For controlling particle shape, higher modulator concentrations can slow down the precipitation rate of amorphous phases, thus improving crystallinity (Fig. 5a).^49–51^ Additionally, modulators physically prevent crystal aggregation, leading to anisotropic growth. This approach provides control over the morphology of the resulting crystals, enabling the formation of smaller, relatively uniform nanoparticles (NPs) in various shapes.^52^ Typically, additives with the same chemical functionality as the linker, such as monocarboxylic acids, are used to control crystal size and morphology. However, other functionalities, such as triethylamine, have also been employed in carboxylate-based MOFs.^50,53^

(ii) pH mediated: higher pH levels shift linker deprotonation equilibria forward, speeding up nucleation rates and influencing morphology.^49,50^ For example, Wang *et al.* reported a change in the morphology of HKUST-1 particles influenced by pH adjustments.^54^ By varying the amounts of sodium formate and triethylamine, they were able to change the pH from 2.23 to 3.88. This shift in pH altered the shape of the synthesized particles (Fig. 5b), transforming them from octahedrons to oval particles.^54^

(iii) Surfactant: amphiphile surfactants adsorb onto crystal facets, altering morphology and controlling particle size (Fig. 5c). The degree of surfactant adsorption depends on its affinity for a specific crystal facet, which is determined by the interaction energy. This selective adsorption can accelerate the growth rate of one facet while decelerating another, affecting how easily reactants can attach to the surface. As a result, surfactant addition influences the shape and size distribution of the final product. Additionally, amphiphiles can aggregate in water and certain solvents to form a soft matrix for nanostructures.^50^ As an example, Lyu *et al.* used cetyltrimethylammonium bromide (CTAB) to produce truncated rhombic dodecahedra (TRD) and rhombic dodecahedra (RD) ZIF-8 particles.^55^

(iv) Solvent-mediated: by adjusting solvent properties, crystal growth and shape can be controlled, affecting nucleation rates, precursor solubility, and crystal formation kinetics.^57^ For example, for TAPB-DMTP COF,^56^ varying PEG-400 to water ratios impacts the COF's morphology and crystallinity (Fig. 5d). At a ratio of H_2_O/PEG-400 = 0.5 mL/2.5 mL, a strong diffraction peak at 2.79° disappeared, indicating disordered imine-linked condensates instead of well-defined COFs. With increasing PEG-400 content, SEM images revealed a shift from solid nanofibrous structures to chrysanthemum-like morphologies, highlighting the importance of solvent composition in tailoring COF properties for diverse applications without high temperatures or harmful solvents.^56^

In their recent review, Zaleska-Medynska *et al.*^50^ cover the strategies and mechanisms for controlling the morphology of synthesized MOF particles. This review covers morphology control of porous materials in a broader context. However, it is worth noting that these mechanisms can still present challenges when aiming to achieve low-dimensional structures such as fibers or nanosheets.^58^ Similar strategies have also been used for COFs. Furthermore, porous materials themselves can serve as highly effective templates for generating porous carbon materials with desired morphologies.^59^ This approach facilitates the creation of diverse structures like carbon nanorods and graphene nanoribbons.

### 2.3. Templated synthesis

Controlling the geometric shape of reticular materials by regulating the bonding behaviour of building blocks is still difficult. A key approach in the synthesis of reticular materials involves the use of templated methods, which are typically categorized into two primary strategies: external templating and internal templating:^60^

**Fig. 6:**
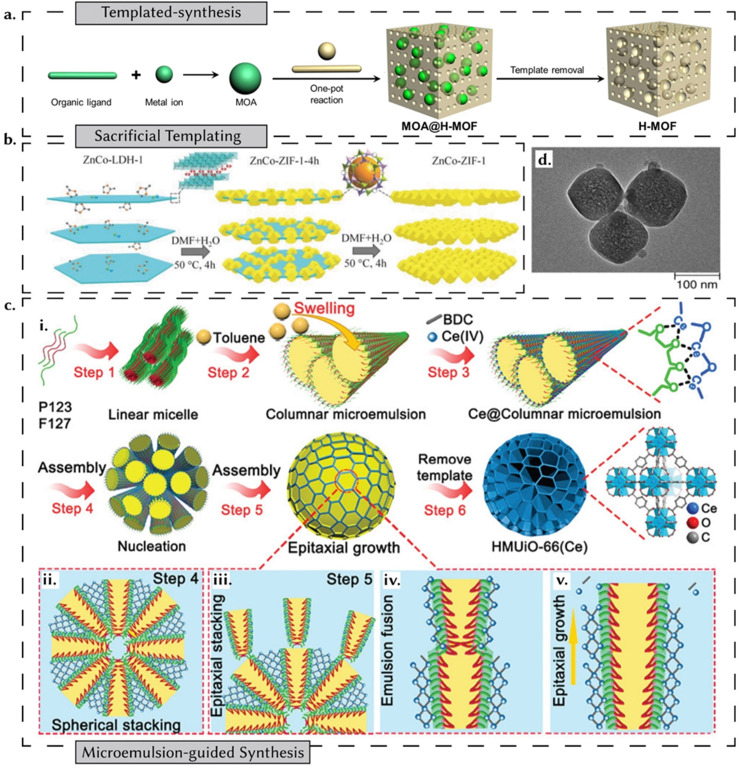
Templated synthesis of reticular materials. (a) H-MOF formed through *in situ* Metal Organic Assembly mediated templated synthesis. Adapted under CC 4.0 license permission from ref. 62. (b) A schematic illustration of two-dimensionally grown bimetallic ZIFs on a sacrificial LDH template. Reprinted with permission from ref. 63. Copyright 2018 Wiley. (c) A schematic illustration of a microemulsion-guided assembly strategy for the synthesis of a cerium-based hierarchically macro-microporous MOF. (i) P123 and F127 (triblock copolymers) act as co-stabilizers and form a columnar microemulsion with toluene as the oil phase. The assembly occurs (ii) spherical stacking and (iii)–(v) epitaxial growth. Reprinted with permission from ref. 64 Copyright 2022 American Chemical Society. (d) TEM image of a UiO-66-based MOF prepared from another MOF acting as a precursor template. Adapted under CC 4.0 license permission from ref. 62.

(i) External templating – more relevant to the focus of this review – uses templates to control the morphology and structure of reticular materials.^60^ The premise of template-assisted synthesis is a relatively straightforward three-step process: the preparation of the template, the synthesis of the desired material on the template, and the subsequent removal of the template (Fig. 6a). Depending on the choice of template, these strategies can be further categorized as hard templating or soft templating – as discussed below.^61^

(ii) Internal templating, or using reticular materials as templates themselves, involves leveraging reticular materials to create other materials while maintaining their original morphology. This strategy allows for precise control over the size, composition, and structure of the derived materials.^60^ For example, ZnO@ZIF-8 nanowires were synthesized using ZIF-8 as a template for growing ZnO, demonstrating the utility of MOFs in fabricating complex nanostructures.^65^ However, since internal templating focuses more on structuring other functional materials rather than the synthesis of reticular materials themselves, it falls outside the scope of this review. Readers seeking more information on both external and internal templating methods are encouraged to explore additional resources.^60^

In hard templating, the templates employed exhibit relative rigidity, essentially acting as structural scaffolds to facilitate the growth of nanostructures with morphologies that complement the template's configuration.^66^ In the case of MOFs, two distinct avenues for harnessing templating strategies have been used. The first involves the employment of templates to guide the synthesis of MOFs with tailored morphologies,^63,67^ while the second entails the utilization of MOFs themselves as templates for the fabrication of complex nanoarchitectures.^68^ In a recent review addressing hard templating strategies for MOFs,^69^ Luque *et al.* categorize strategies into three distinct groups: (i) sacrificial, wherein templates are dissolved or removed after MOF synthesis; (ii) semi-sacrificial, characterized by templates that are neither entirely eliminated nor fully retained in the final MOF structure; and (iii) non-sacrificial, where templates persist after synthesis, giving rise to template-MOF composite materials. Within sacrificial templating strategies, hard templates, such as alumina, silica, and polystyrene spheres, offer a more stable approach.^70,71^ These rigid scaffolds guide the growth of reticular materials into specific shapes.^58^ For instance, PS@ZIF-8 composites can be produced by embedding polystyrene spheres in the synthesis mixture and then removing them, resulting in hollow ZIF-8 structures.^72^ Also, silica templates offer a finer degree of control over shape, porosity, and surface area and can be conveniently functionalized to enable the synthesis of multifunctional MOFs.^69^ SBA-15 mesoporous silica has been used as a template to grow MOF-5, illustrating how template structures can significantly influence MOF formation and properties.^73^ However, MOFs need to exhibit resistance to hydrofluoric acid (HF) when employing silica as a sacrificial template, as HF is the sole viable etching agent for silica. For two-dimensionally grown MOFs, layered double hydroxides (LDHs), composed of positively charged layers interspersed with anionic regions between them, prove effective as sacrificial templates, as illustrated in the case of bimetallic Zn–Co ZIFs (Fig. 6b).^69^ Following this strategy of taking advantage of the different chemical stabilities of MOF precursors, metal–organic assemblies (MOAs) have been used as a template for the synthesis of a hierarchical-pore MOF (H-MOF) (Fig. 6a).^62^ An example of semi-sacrificial templates is illustrated by metal oxides. In the presence of appropriate organic linkers, surface metal oxide entities transform, leading to the formation of corresponding MOFs.^74^ Through the manipulation of linker quantities and reaction durations, the extent of this conversion can be regulated, ultimately yielding metal oxide-MOF composite materials. When a metal oxide composite is used as a template, the partial degradation of the metal oxide fraction of the template yields yolk–shell structures like Pd@ZIF-8.^75^ The most extensive category of templates is non-sacrificial. These templates encompass a wide array of materials, including polymers, MOF-based structures, silica templates (spherical and mesoporous), noble metals, metal oxides, CNTs, LDHs, and zeolites.

For hard templates, material–template interactions must be tailored. In this context, various strategies have been devised, including surface interactions with the template, the electrostatic assembly of negatively charged shell materials onto positively charged cores,^76^ and techniques such as chemical vapour deposition (CVD)^77^ and atomic layer deposition (ALD).^78^ The main limitation of hard templating is the availability of templates with suitable dimensions, shapes, and surface characteristics. Furthermore, the high associated costs often render such techniques impractical for large-scale applications. In contrast, soft templating strategies hold promise for creating hierarchically porous MOFs. However, the synthesis conditions for MOFs may not facilitate the self-assembly of soft templates. Additionally, achieving mesopore formation post-template removal poses challenges, mainly due to phase separation. Phase separation occurs when components in a mixture separate into distinct phases, potentially disrupting the uniform pore distribution in the final MOF structure.^67^ To mitigate these challenges, certain strategies have been developed, such as the introduction of Hofmeister ions.^67,79^ This approach, termed salting-in ion-mediated self-assembly (SIMS), promotes the self-assembly of MOFs under relatively milder conditions.^79^ Typically, this includes ionic surfactants, nonionic copolymers, and emulsions (Fig. 6c).^64,80,81^ Soft templates, such as CTAB and triblock copolymers, are employed to create hierarchical porous MOFs by guiding the structure during synthesis. For example, CTAB has been used effectively to synthesize mesoporous MOFs with hierarchical porosity. However, the stability of small molecular micelles can be a limitation of this approach. In the case of using MOFs as templates for the synthesis of secondary MOFs, the approach leads to the generation of hierarchically porous structures with mesoporous sizes controllable by regulating template quantity (Fig. 6d).^58^ Conversely, template MOFs may serve as seeds for the epitaxial growth of secondary MOFs with new linkers, leading to core–shell MOF composites.^58,82^ Again, these principles of templating have been further translated to the structuring of other classes of porous materials like COFs^83–86^ and POCs.^87^

### 2.4. Self-assembled superstructures

**Fig. 7:**
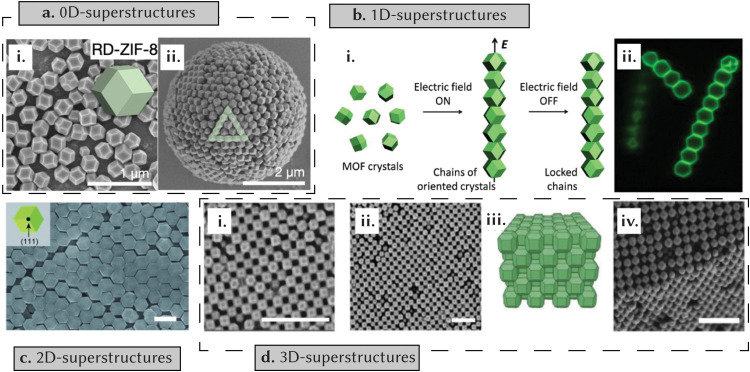
The self-assembly of superstructures. (a) Solvent evaporation from the surface of emulsion droplets results in the formation of a rhombic dodecahedral 0D ZIF-8 superstructure. (i) SEM image of monodisperse particles, (ii) SEM image of assembled superstructure. Reprinted with permission from ref. 89 Copyright 2022 Wiley. (b) Surface polarization of the electrostatic double layer leads to the assembly of 1D chains of ZIF-8 crystals. (i) A schematic illustration of the process, (ii) confocal cross-sections perpendicular to the applied field. Reprinted with permission from ref. 90 Copyright 2013 American Chemical Society. (c) Formation of 2D superstructures achieved through the gradual evaporation of MOF particle colloidal solutions. SEM image of the superstructure (scale = 1 μm). Reprinted with permission from ref. 91 Copyright 2012 Wiley. (d) Surfactant-mediated orientation results in 3D MOF superstructures. (i) and (ii) SEM images depict the packing of MOF crystals in the superstructure (scale = 2 μm, 1 μm respectively). (iii) Schematic illustration of packing. (iv) SEM image of the crystal. Reprinted with permission from ref. 94 Copyright 2019 Wiley.

Porous reticular materials have been assembled as 0, 1, 2 and 3D-ordered superstructures. 0D superstructures typically form in tightly confined nano- or micro-spaces, often through methods such as solvent evaporation or spray drying.^88^ For example, Vogel *et al.* used solvent evaporation from the surface of emulsion droplets to achieve diverse morphologies of ZIF-8 (such as cubic, rhombic dodecahedral, and truncated rhombic dodecahedral) and octahedral UiO-66 as self-assembled superstructures (Fig. 7a).^89^ Moving to 1D superstructures, induction of 1D self-assembly of particles often necessitates the use of driving forces such as electric and magnetic fields. Granick *et al.* capitalized on the surface polarization of the electrostatic double layer of ZIF-8 particles, dispersing them in ethylene glycol and applying an AC electric field to generate 1D chains.^90^ This assembly arose from particle-to-particle contact driven by dipole–dipole interactions (Fig. 7b). Formation of 2D superstructures has been done through the gradual evaporation of colloidal solutions of MOF particles (Fig. 7c),^91^ the spreading of colloidal solutions across air–liquid interfaces with subsequent interface modification to drive assembly,^92^ and the surface modification of MOFs using oligonucleotides, capitalizing on associated hydrogen bonding.^88^ In turn, the creation of 3D superstructures has been achieved through sedimentation methods,^92^ solvent evaporation^93^ and the use of specifically tailored surfactants (Fig. 7d).^94^ Moving to COFs, relatively few comprehensive studies have focused on mechanisms governing self-assembly. An early investigation in this context was from Banerjee *et al.*,^84^ who studied the self-assembly mechanisms of hollow-sphere COFs. Their approach involved isolating samples at fixed intervals during the initial reaction and subjecting them to detailed analyses using scanning electron microscopy (SEM), transmission electron microscopy (TEM) and atomic force microscopy (AFM). At 12 h, they found rod-like morphologies, with crystallites forming through π–π stacking of COF layers. As the reaction progressed to 24 h, these crystallites began to aggregate, forming a spherical morphology with hollow cavities forming inside after 36 h of reaction time. At 48 and 72 h, the surfaces of these structures smoothened out due to the fusion of crystallites. Similar in-depth analyses have been done to study the self-assembly mechanisms governing microtubular COFs.^95^

## 3. The macroscopic structuring landscape

In the last ten years, there has been a big push in research to figure out and use different ways to shape and structure porous materials such as MOFs.^96^ Here, we discuss the various shapes and structures one can use to get the best performance in industrial settings. The central idea is to keep the material's core properties intact from the tiny, microscopic level all the way up to the larger, macroscopic scale to ensure it works just as well in real-world applications. With a whole array of macroscopic forms to consider – such as granules, pellets, self-assembled monoliths, thin films, gels, foams, and glasses – picking the right shape gets more complex. The choice of shape is largely dictated by the application requirements. For instance, in gas storage applications, given fast kinetics, the most important metric is – arguably – the volumetric capacity of the adsorbent. Here, powdery materials with low bulk density will perform poorly, whereas dense monoliths will be the go-to choice. Moreover, due to the nature of the application, the adsorbent is subject to a high degree of mechanical stress during handling and operation, demanding high mechanical strength. In such cases, monoliths fabricated through a sol–gel route are promising candidates since they provide the native porosity of the porous structure but facilitate excellent mechanical properties and particle densities close to single-crystal ones.^18^ In molecular sieving-based applications – such as gas separations and water decontamination^97^ – pore sizes, chemical stability, tensile strength and flexibility are of primary importance – wherein membranes can be ideal candidates^33,98^ – although high density is still of major importance.

Broadly speaking, the industrial applications covered here are directly linked to the adsorption properties of the porous materials, and thus their performance will be dictated by properties such as adsorption uptake, selectivity, adsorption kinetics, heat of adsorption, heat conductivity, and stability – with cost being another important question. Importantly, the choice of shaping process has an impact on these properties. For example, the use of binders might partially block the porosity and reduce the adsorption uptake,^99^ while the application of mechanical pressure may partially collapse the porosity. Adsorption kinetics are particularly relevant and often ignored since, again, the shape factor is critical. Here, techniques leading to thicker-shaped materials might result in longer diffusion pathways, which would reduce the efficiency of the material's performance. At the same time, a lack of densification might reduce the density of the final material and, hence, the volumetric adsorption capacity as well as the heat conductivity, key for heat dissipation during the exothermic adsorption process.^100^ Looking back at binders, a poor selection might result in the creation of microstructures with reduced thermal conductivity compared to the original bulk material.

Stability is another crucial consideration – which has been the focus of several excellent reviews^33,101^ – and thus is discussed in the present context very briefly. Here, stability takes different forms – mainly thermal, chemical, and mechanical. The ability to withstand relatively high temperatures without undergoing significant structural or property changes is crucial for the success and reliability of shaped materials.^102,103^ Chemical stability is the ability of shaped materials to remain chemically stable in the presence of reactive gases, corrosive substances, acidic or basic environments.^102,104^ Mechanical stability ensures that the shaped materials can withstand mechanical stresses due to *e.g.*, moves, vibrations, and the weight of a packed bed and maintain their structural integrity during handling, installation, and use, ultimately enhancing their reliability and longevity in real-world applications.

**Fig. 8:**
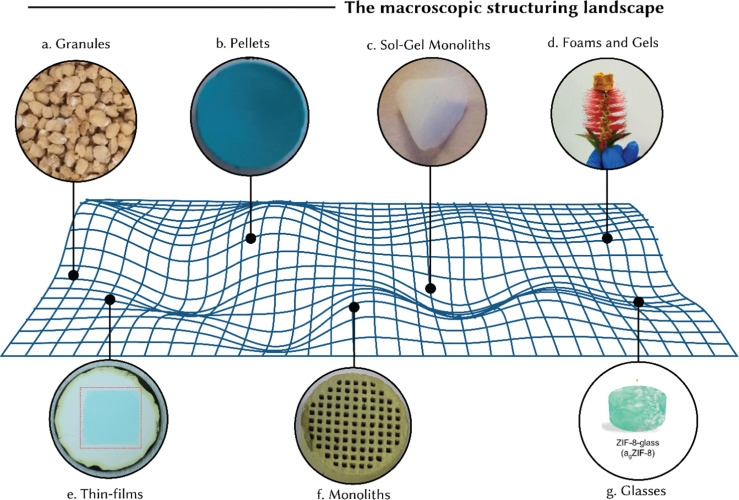
Different macroscopic forms of reticular materials: (a) granules: MIL-88B(Fe) granules, reprinted with permission from ref. 105 Copyright 2022 Elsevier. (b) Pellets: HKUST-1 pellets, reprinted with permission from ref. 106 Copyright 2020 MDPI. (c) Sol–gel monoliths: UiO-66 monoliths formed using a sol–gel route, reprinted with permission from ref. 107 Copyright 2019 Springer Nature. (d) Foams and gels: MOP/COF composite aerogel, reprinted with permission from ref. 108 Copyright 2024 Springer Nature. (e) Thin-films: wrinkled MOF thin-film with Turing patterns on a porous alumina support, reprinted with permission from ref. 109 Copyright 2024 American Association for the Advancement of Science. (f) Monoliths: MIL-101(Cr) monolith structured using an extrusion-based method, reprinted with permission from ref. 110 Copyright 2020 Elsevier. (g) Glasses: schematic visualisation of a ZIF-8 glass, reprinted with permission from ref. 111 Copyright 2024 Springer Nature.

Although the underlying principles governing reticular material stability are universal, application-specific considerations need to be investigated. We present here a discussion on various shapes including granules, pellets, monoliths, sol–gel monoliths, foams and gels, thin films, and glasses (Fig. 8). In turn, Section 4 will detail the structuring techniques utilized for their formation, providing case studies that illustrate the implementation of structuring techniques to produce reticular materials.

### 3.1. Granules

Granular materials (Fig. 8a) are generic shapes used in industry as they are relatively easy to translate to large scales at relatively lower costs.^112^ These solid bodies come in various sizes and associated particle size distributions,^113^ Their flexibility facilitates the customization of these structures to meet the requirements of different processes and industries. In particular, granules are used in packed beds and fluidized bed reactors, where the particle size distribution enhances heat and mass transfer.^114^ At the same time, the particle size and packing density of the granules impact their flowability,^115^*i.e.* the ability of the powder or granules to fall or flow over the influence of their weight. In general, high flowability is desired because it eases the handling and processing of the materials, something that is achieved using large particle sizes. On the other hand, large particle sizes reduce the adsorption kinetics and, therefore, the optimal granule size depends on the final engineering system. Typically, these structures must withstand immense pressures and associated crushing forces throughout their application and, therefore, their mechanical properties are critical. Crucially, granules can retain the porosity of the material obtained at the microscopic scale, with acceptable levels of drops in the porosity upon structuring.

MOFs shaped as granular composites have demonstrated good capabilities in terms of adsorption performance. For example, granular composites of MIL-100(Fe) loaded onto an alumina support demonstrated enhanced performance for tetracycline hydrochloride adsorption when compared to bare MOF powder.^116^ The composite exhibited an adsorption efficiency of 95% within 60 minutes, even with low MOF loading (approximately 3 wt%), compared to both activated Al_2_O_3_ alone (51%) and MIL-100(Fe) powder alone (72%). Additionally, the results revealed a broad pH applicability range (pH 4 to 10) for the composites, with minimal influence from most inorganic ions in solution, apart from fulvic acid and carbonate. Moreover, the composites displayed excellent operability, recyclability, and regenerability through photolysis. The superior adsorptive performance of the granular MOF composites was attributed to synergistic interactions between the MOF layer and activated Al_2_O_3_ support. The successful loading of MIL-68(Al) onto activated Al_2_O_3_ further confirmed the efficacy of the synthesis route.^116^

### 3.2. Pellets

Pellets are small, typically cylindrical, or spherical solid particles (Fig. 8b).^106^ Compared to granules, pellets have narrower particle size distributions, achieved through more precise manufacturing techniques, typically extrusion and spheronization. Sometimes, MOF powders can be tableted, providing morphologies similar to pellets.^117^ Uniformity in size and shape is particularly important in packed bed configurations, as it ensures consistent flow patterns and minimizes pressure drop variations.^118^ More than granules, pellets are engineered for high mechanical strength and crush resistance, essential for withstanding the rigors of handling, transport, and use in industrial settings. Pellets can endure high mechanical pressures and stresses, ensuring they maintain their structural integrity throughout the duration of a process.^119^ For example, pellets of HKUST-1 have been prepared using extrusion – an industrially scalable process – where water is added to the MOF powder to form a paste, which is then heated in a syringe (Fig. 8b).^106^ Using sucrose as a binder, a Zr-based MOF powder has been shaped into spherical pellets with diameters ranging 0.5 to 15 mm in a kilogram-scale batch.^120^ These Zr-MOF pellets showed no mechanical degradation after 70 consecutive drops from a 0.5-meter height, while approximately 5% of the pellets fractured during a 60-minute tumbling test at 25 rpm. In this example, however, it should be noted that there was a nearly 50% reduction in surface area, resulting in just 60% of the original hydrogen storage capacity of the powdered MOF (*i.e.* 1.54 wt% measured at 77 K and 1 bar). The volumetric capacities of the pellets cannot be compared as there is no density reported for the synthesized and shaped materials.^120^

### 3.3. Monoliths

The IUPAC defines a monolith as a shaped, fabricated body with a homogenous microstructure that does not exhibit any structural components that can be distinguished by optical microscopy.^121^ Going by this generic definition, many shapes being discussed in this review can be considered monoliths. In industry, however, monoliths are seen as structured beds featuring large channels – straight or a labyrinthine network (Fig. 8f). The channels' size and connectivity are essential as they promote fluid and reactant flow and ensure efficient mass transport while minimizing flow resistance and pressure drop. Monoliths provide high pore volume and external surface area, essential to promote active sites for adsorption and catalysis.^122^ Typical examples include honeycomb monoliths used in catalytic converters. Here, channel connectivity enables reactant access to catalytic sites, influencing reaction rates.^123^ Monoliths enable fast kinetics and are engineered for structural stability and mechanical strength – characteristics vital for long-term use in the demanding environments where they are used. They can withstand variations in pressure, flow rates, and temperature, making them suitable for continuous processes in industries such as petrochemicals and environmental remediation.^124^ Importantly, a monolithic, structured bed can be coated by an active phase for adsorption or catalytic activity. As an example, Darunte *et al.* demonstrated that honeycomb monoliths supported with mmen-Mg_2_(dobpdc) exhibited a CO_2_ uptake of 2.37 mmol g^−1^ and excellent cyclic adsorption/desorption performance.^125^ The monoliths had MgO and Mg_2_(dobpdc) loadings of 8–10% and 14–18%, respectively. Despite being tested on 100 cells per square inch (cpsi) monoliths, it is anticipated that utilizing dimensional differences could lead to 2–3 times higher weight loading on 400 or 600 cpsi monoliths. Further optimization could result in different MgO loadings on the monolith, affecting the transformation rate from MgO to Mg_2_(dobpdc). Nitrogen isotherms showed significant microporosity after growth, with N_2_ physisorption uptake reaching 58.5% of Mg_2_(dobpdc) in powder form at *P*/*P*_0_ = 0.6. CO_2_ adsorption capacity was 2.37 mmol g^−1^ at 10% CO_2_ concentration in helium and 2.88 mmol g^−1^ using pure CO_2_, with film-form mmen-Mg_2_(dobpdc) achieving approximately 70% of powder-form uptake at 10% CO_2_ concentration.^125^

Along similar lines, Lawson *et al.*^126^ investigated the immobilization of MOF-74(Ni) and UTSA-16(Co) on commercial cordierite monoliths (600 cpsi) for CO_2_ capture. MOF-74(Ni)- and UTSA-16(Co)-cordierite monoliths with loadings as high as 52% and 55%, respectively, were prepared using layer-by-layer + secondary growth and *in situ* dip coating techniques. Both methods produced uniform MOF layers on the cordierite surface. The layer-by-layer plus secondary growth method showed promise for MOF-74(Ni) growth, while *in situ* dip coating yielded thick UTSA-16(Co) layers. They further improved this process by incorporating the MOFs on a polyamide-imide Torlon monolith using the existing carbon hollow fiber surface. Prior to MOF growth, the carbon hollow fibers were functionalized with hydroxyl groups to improve their integration. They then used dip-coating and layer-by-layer techniques to grow MOFs. The composites exhibited loadings of 38 wt% of MOF and film thicknesses ranging 10 to 15 mm and surface areas of 266 and 211 m^2^ g^−1^ for MOF-74/carbon and UTSA-16/carbon composites, respectively, along with pore volumes of 0.28 and 0.20 cm^3^ g^−1^. As proof of concept, they reached CO_2_ adsorption capacities of 1.2 and 2.0 mmol g^−1^ for MOF-74 and UTSA-16 composites, respectively, at room temperature and 1 bar. Looking ahead, alternative methods may be explored to refine the coating procedure and thereby, improve film growth on carbon hollow fibers, with the goal of mitigating the reduction in BET area associated with strong acids.^126^

### 3.4. Sol–gel monoliths

**Fig. 9:**
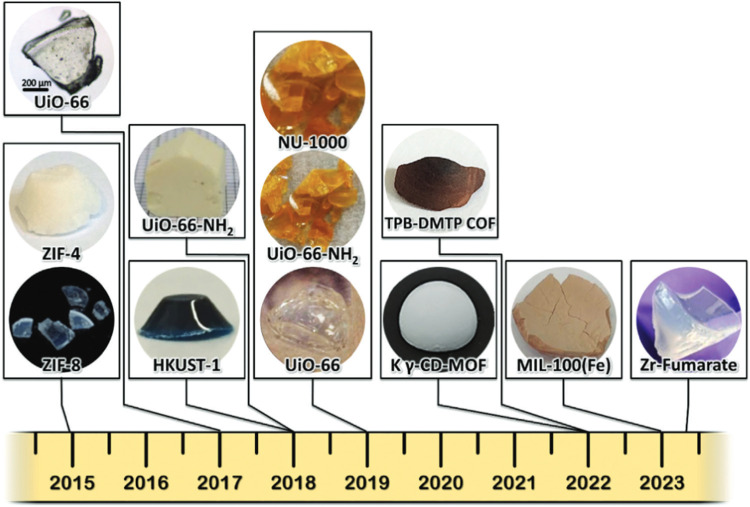
Chronological overview of sol–gel monoliths, accompanied by optical images, illustrating the self-shaping evolution of key monolithic MOFs and COFs. Reproduced with permission from ref. 127 Copyright 2023 Wiley.

In addition to the conventional definition, the term ‘monolith’ is used many times to describe a single, continuous structure without joints or seams, often characterized by a uniform material composition throughout (Fig. 8c). This definition emphasizes the monolithic nature as a single piece. The term ‘monolith’ describes, therefore, single bodies of shaped materials formed during synthesis, which can lead to either densified materials, where the adsorbent's volumetric capacity is crucial^127^ but also ultralight ones.^128,129^ These shaped materials often resemble foams, aerogels and xerogels described in Section 3.5, and are, frequently, referred to by these names in the literature. Arguably, foams and gels can be monoliths but only receive this name when they are structured as a single body rather than a powder.^99^ Monolithic adsorbents are, for example, used in chromatography and benefit from their large surface area and macro-/mesoporous network, enabling efficient separation of compounds in complex mixtures.^130^ Over the past few years, various analogues of sol–gel monolithic MOFs and COFs have been synthesized and reported in the literature^127^ (Fig. 9), beginning with the ZIF-8 monolithic synthesis reported by Tian *et al.* in 2015.^131^

Sol–gel materials have been studied for a range of energy-related applications, particularly in gas storage, where they have displayed record performances due to their high density and volumetric adsorption capacities.^16,18^ A notable example of such monoliths in gas storage applications is the use of HKUST-1 for H_2_ storage.^18^ The relatively high density of monoliths enhances their volumetric adsorption properties, rendering them advantageous for diverse uses. Unlike conventional structures, sol–gel monoliths do not require binders in their formation, resulting in a more uniform and pure material composition. An additional advantage lies in the precise control over their micro/mesoporosity achievable by manipulating synthesis parameters.^107^ This level of control facilitates the creation of tailored pore structures, allowing for the fine adjustment of properties to suit specific application needs.

### 3.5. Foams and gels

**Fig. 10:**
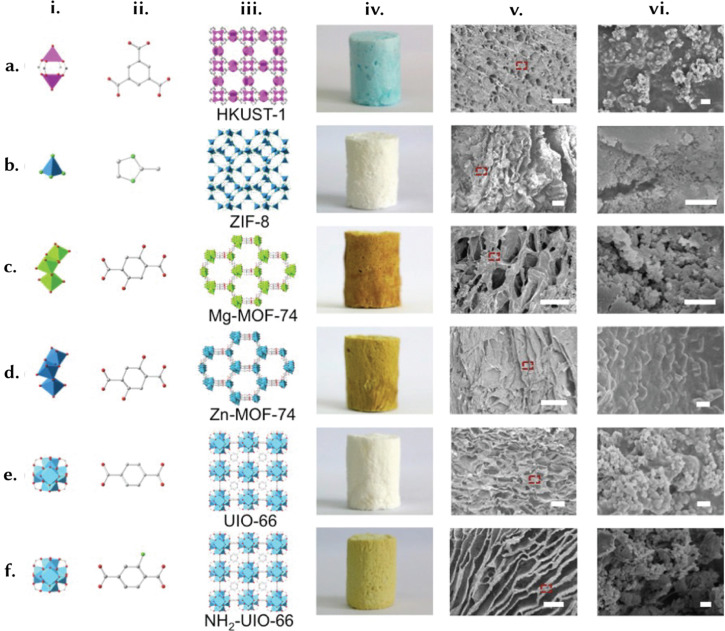
Shaping different MOF structures into foams. Foams are materials containing gaseous voids surrounded by a denser matrix. Some gel stages can also be technically classified as foams. The defining feature of such structures is high porosity, characterised by a significant volume of pores and voids. To illustrate the concept of these structured materials, we present (i) and (ii) the precursors, (iii) crystal structures, (iv) optical images, and (v) and (vi) SEM images with a scale of 100 and 1 μm, respectively, for (a) HKUST-1, (b) ZIF-8, (c) Mg-MOF-74, (d) Zn-MOF-74, (e) UiO-66, and (f) NH_2_-UiO-66. Adapted with permission from ref. 135 Copyright 2016 American Chemical Society.

Foams consist of empty macropores encased within a denser matrix or skeleton. In contrast, a gel is a non-fluid, colloidal network saturated with a liquid. When the space within a gel is evacuated and replaced by a gas, preventing minimal structural change, it transforms into an aerogel (Fig. 8d). Conversely, reducing this space results in the formation of a xerogel.^132^ Based on their composition, cell morphology, and physical properties, polymer foams can be categorised into two types: rigid or flexible. Depending on the size of the foam cells, they can be further classified as macrocellular (larger than 100 μm), microcellular (ranging from 1 to 100 μm), ultra-microcellular (0.1 to 1 μm), and nanocellular (0.1 to 100 nm). Furthermore, polymer foams can be classified as either open-cell or closed-cell. Closed-cell foams are characterised by isolated voids and cavities surrounded by cell walls. In contrast, open-cell foams possess broken cell walls and primarily consist of ribs and struts.^133^ The defining feature of both foams and gels is their high porosity, marked by a substantial volume of voids or pores.^134^ For instance, Chen *et al.* demonstrated a continuous phase transformation strategy to shape MOFs from processable fluids (Fig. 10).^135^ They presented a cup-shaped Cu–MOF composite based on HKUST-1 and a hierarchically porous MOF foam with high catalytic efficiency in C–H oxidation, achieving 76% conversion and 93% selectivity for the composite, and 92% conversion and 97% selectivity for the foam, along with improved recycling and kinetics. This procedure was extended to ZIF-8, Mg-MOF-74, Zn-MOF-74, UiO-66, and NH_2_-UiO-66. These low-density MOF-based foams (<0.1 g cm^−3^) with high MOF loadings (up to 80 wt%) featured hierarchically porous structures and uniformly distributed, fully accessible MOFs. These foams exhibited a low energy penalty (pressure drop <20 Pa at 500 mL min^−1^) and, as a result, can be employed as membrane reactors.

### 3.6. Thin films

A thin film is a layer that extends in two dimensions but the thickness ranges from several nanometers to a few micrometers and are designed for use as coatings on a substrate (Fig. 8e). Their thickness is controlled to meet specific needs; precision in this context is vital, as slight variations in thickness can have an important influence on their performance.^136^ Moreover, thin films must maintain uniformity across the entire surface to ensure consistent functionality. Additionally, the surface of thin films can be finely tuned to achieve controlled roughness, which impacts properties such as adhesion, friction, and wettability, thereby facilitating tailored interactions with other materials.^136^ For instance, Luo *et al.* introduced an innovative synthesis method to produce wrinkled thin films of HKUST-1 with Turing patterns (Fig. 8e), successfully balancing high MOF loading with mechanical flexibility.^109^ By employing a confined interfacial reaction between a zinc oxide thin film and a polymer topcoat, they achieved wrinkle configurations that enhance strain tolerance up to 53.2% and significantly increase the surface area, reaching a BET area of 1473 m^2^ g^−1^. The films displayed a notable MOF loading of 96.3 wt% and formed 13 distinct Turing patterns through precise adjustments of reagent concentrations and polymer thickness. In practical applications, these films demonstrated impressive hydrogen permeance of up to 6.82 × 10^4^ gas permeation unit (GPU) and H_2_/CO_2_ selectivity of 15.3. When stacked, the films achieved even higher selectivity (41.2) and permeance (8.46 × 10^3^ GPU). Furthermore, the films maintained their structural integrity during transfer onto various substrates and exhibited a high elongation at break of 41.6%, highlighting their potential for use in flexible electronics and efficient gas separation membranes.^109^

**Fig. 11:**
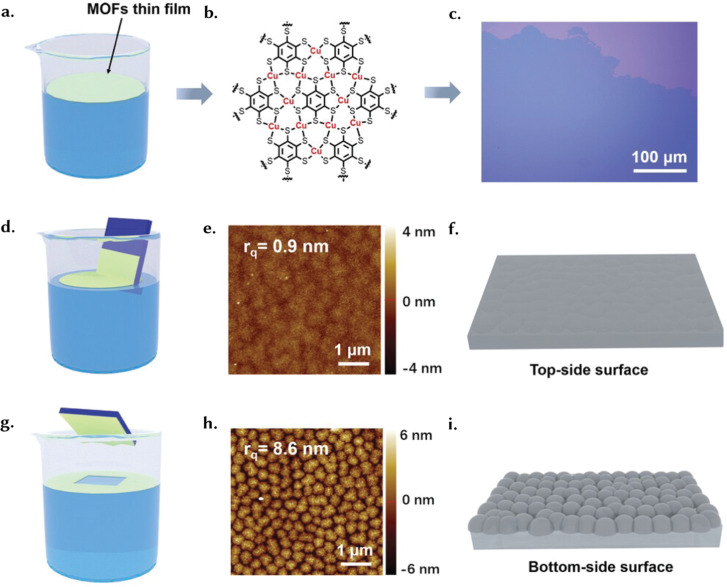
(a) A schematic illustration of the gas–liquid interfacial reaction method utilised to produce Cu-BHT films. (b) A schematic of the structure of the prepared Cu-BHT film. (c) Optical microscopy images of the prepared film. (d) A schematic illustration of the transfer method for obtaining the flat upper-side surface. (e) AFM images of the flat upper-side surface. (f) A schematic illustration of the flat upper-side surface of the Cu-BHT film. (g) A schematic illustration of the transfer method for obtaining the bottom-side surface. (h) AFM images of the bottom-side surface. (i) A schematic illustration of the bottom-side surface – with synaptic-like structures of the Cu-BHT film. Reprinted with permission from ref. 137 Copyright 2021 Wiley.

Surface roughness and morphology can also impact sensing and biosensing performance. For example, Chen *et al.* fabricated on-chip electrochemical micro-biosensors using an electrically conductive Cu-benzenehexathiol (Cu-BHT) film (Fig. 11a), with a flat upper surface (Fig. 11b) and an undulating bottom surface (Fig. 11c).^137^ The bottom surface, with dense crystal defects (ts-Cu) acting as nanozymes, showed higher H_2_O_2_ sensing performance than the smoother upper surface. Crystal defects can enhance the electrocatalytic interfaces, typically buried between the solid support and liquid electrolyte in conventional sensing methods. Thin films can be tailored based on the material used and so can be engineered with precision to achieve specific pore size distributions, typically within the range of micropores.^138^ This is of course relevant for applications such as filtration or controlled diffusion, where selective separation and diffusion is a primary requirement.^139–141^

### 3.7. Glasses

While the discussion thus far has predominantly centred on solid, crystalline reticular materials, recent explorations into the order–disorder dynamics and solid–liquid–glass transitions within certain MOFs have shown the possibility of creating glassy, non-crystalline materials (Fig. 8g). Here, it is important to draw a distinction between amorphous MOFs and MOF glasses. Ma and Horike define a ‘glass’ as an ‘undercooled frozen-in liquid’.^142^ For an amorphous MOF to be classified as a ‘glass’ according to this definition, there needs to be an absence of long-range atomic ordering and a second-order phase transition to a soft liquid-like state at the glass-transition temperature (*T*_g_). For more theoretical insights, we refer readers to focused reviews dedicated to MOF glasses.^142,143^ In principle and given that a material does not lose its intrinsic properties such as porosity, melting a material is potentially the most effective way of implementing shaping and densification. However, and despite their recent interest, the transition from crystalline to glassy phases often results in the loss of the innate porous structure of coordination polymers,^34^ limiting its impact in conventional applications such as gas storage, separation, and catalysis. Consequently, glassy materials may face challenges when competing with porous counterparts due to the lack or reduced porosity.^144^ On the other hand, glassy MOFs – like glassy carbons before – lack grain boundaries, enhancing their mechanical stability and, for conductive MOFs, their conductivity, while their flexibility enables easier manipulation. This makes them good candidates for various applications, particularly as electrodes,^145^ electrolytes,^146^ or membranes.^147^

## 4. Structuring of reticular materials at the macroscopic scale

While there have been several developments with regards to ‘novel’ techniques for achieving the shapes introduced so far, there are complexities that need to be considered for industrial translation. These considerations are many times related to aspects of process engineering more than to the chemistry of the materials. Important considerations include production throughput – *i.e.*, the amount of product that is produced in terms of the space-time yield,^96^ ‘quality’ of the final product, and cost of the overall process – *i.e.*, cost of reagents, human power, utility, and transportation, among others. In most applications, it is necessary to reach a compromise involving these aspects. For instance, in applications where products of high-quality may be required (*e.g.*, sensing), the production throughput may be low, and the costs may be high. Conversely, applications where high throughput and low cost is desired may suffer from lower product quality. In this section we offer insights into the techniques available for achieving the diverse landscape of shapes discussed in Section 3. We classify these techniques into two major categories as *in situ* shaping and post-synthetic shaping. *in situ* shaping techniques involve the synthesis and structuring of materials simultaneously in a single step, offering precise control over the final shape and structure. Examples include sol–gel synthesis, dip-coating, deposition techniques, and spray drying. Conversely, post-synthetic shaping techniques involve shaping of the already synthesized materials, mostly in the powder form. This category encompasses methods like pelletization, granulation, extrusion, spheronization, 3D printing, phase inversion, hydrogelation, and glass formation, allowing greater flexibility in shaping materials after synthesis, thereby enabling the production of complex geometries and tailored structures.

### 4.1. *In situ* shaping

#### 4.1.1. Sol–gel monolith synthesis

**Fig. 12:**
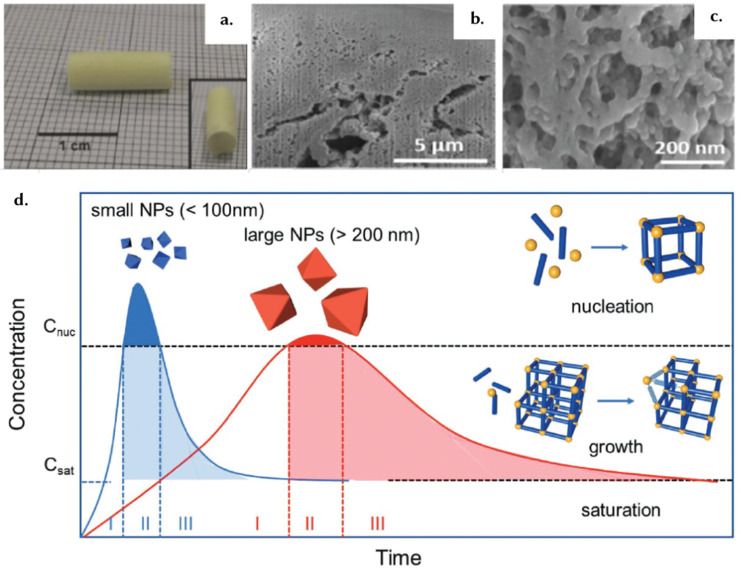
The self-assembly of superstructures. (a) The image showcases the solid and mechanically sturdy macrostructure of a Zr-based MOF, UiO-66-NH_2_, obtained through the supercritical CO_2_ drying technique from the initial gel state. This macrostructure effectively retains the intricate features and inherent characteristics of the microstructure, as evidenced by the SEM micrographs presented in (b) and (c). Reprinted with permission from ref. 149 Copyright 2018 Royal Society of Chemistry. (d) A schematic representation of MOF NP nucleation and growth based on the LaMer model. The blue trace illustrates that the synthesis of uniform small MOF NPs involves a rapid formation of numerous, abundant nuclei. In contrast, the red trace shows that a limited number of nuclei and a slower growth rate lead to the formation of uniform large NPs. Reproduced with permission from ref. 150 Copyright 2018, John Wiley and Sons.

From a mechanistic standpoint, the formation and packing of the primary particles of a sol–gel during the synthesis procedure governs the macroporous structure and macroscopic properties.^127^ An example of this is shown in Fig. 9 and 12a, with cm-sized monoliths. These monoliths result from the very small NPs formed during the sol–gel process, contributing to the formation of a gel product (Fig. 12b and c). Self-assembly of primary particles in sol–gel monoliths occurs through a sequence of well-defined processes, including supersaturation, nucleation, growth, and Ostwald ripening (Fig. 12d).^127,148^ During the nucleation stage, the MOF seeds, or nuclei, act as substrates for subsequent crystal growth. During the growth stage, the interplay of external surface reactions and diffusion of monomers becomes important. During the subsequent Ostwald ripening stage, larger particles grow at the expense of smaller particles.^40^ Finally, during the drying process of the sol–gel synthesis, the gradual solvent removal increases the likelihood of nanoparticle agglomeration into bulk monolithic structures.^99^ Overall, controlling particle size during sol–gel synthesis is crucial for the formation of monolithic structures, emphasizing the importance of employing appropriate techniques for size control during the sol–gel reaction to yield optimal structures.^127^

Controlling the kinetics of material formation is a fundamental method for regulating particle growth. By shortening the reaction time, starting crystals have less time to grow, resulting in smaller primary particles. However, this can result in a lower yield and consequently, reduced crystallinity and surface area due to unreacted precursors.^127^ Temperature is another crucial factor in crystallization kinetics; lowering the synthesis temperature slows down the reaction, resulting in smaller primary particles.^151,152^ However, altering temperature can have complex effects on the chemical reaction. Additionally, not all materials can be synthesized at lower temperatures, as some reactions require a minimum temperature to proceed. For instance, while ZIF-8^131^ and RT-COF-1^153^ can be synthesized at room temperature, UiO-66 requires 100 °C.^107^ Upon achieving a gel of primary particles, obtaining a monolith involves several steps. Firstly, centrifugation is used to separate the material from the reaction media. The speed at which centrifugation is performed plays a critical role in the formation of the monolith. Higher centrifugation speeds lead to stronger centrifugal forces acting on the particles, causing them to settle more rapidly. Consequently, faster centrifugation rates typically result in better compaction of primary particles, leading to denser monoliths. This is because the increased centrifugal force helps to pack the particles more closely together, reducing the void spaces between them. As a result, the final monolith exhibits enhanced structural integrity and mechanical strength. Conversely, lower centrifugation speeds may not provide sufficient force to compact the particles effectively, resulting in a less dense and weaker monolith.^127^

Following centrifugation, the drying phase emerges as a critical step, demanding customized approaches tailored to each material type.^127^ Typically, slower drying processes are preferable for monolith formation over powder formation. Material-specific considerations come to the forefront during this stage; while for many MOFs may suffice with room temperature drying, rigid COFs often necessitate supercritical CO_2_ (scCO_2_) drying to avoid the meniscus and capillary forces in the gas–liquid interface when evaporating the solvent. For example, in the case of TPB-DMTP-COF pellets, higher acetonitrile fractions result in a sharp decline in the BET area due to pore disruption induced by capillary action. To address this issue, a sample processed in pure acetonitrile and activated in scCO_2_ not only restored full porosity but also exhibited a BET area consistent with the desired trend. This offers crucial insights into the efficacy of scCO_2_ treatment in mitigating pore damage and preserving porosity and surface area. Moreover, fine-tuning of the BET area was achieved by adjusting the rate of scCO_2_ pressure release, further affirming the importance of optimal supercritical activation in maintaining material integrity.^154^ Another instance of material-specific treatment is evident in Cu-centered MOF (_mono_HKUST-1) monolith formation, where the drying temperature is dictated by particle size. Larger particles necessitate lower drying temperatures to achieve a monolith, while smaller ones can withstand higher temperatures for monolith formation. For example, particles with a size of 51 nm can be dried at 40 °C to achieve a monolithic structure, whereas those with a size of 73 nm can only endure drying at 30 °C. Conversely, particles sized at 145 nm fail to yield a monolithic structure at any temperature, attributed to mechanical stress induced by solvent surface tension.^18^ A similar strategy allowed us to produce monoliths for Zr-based UiO-66 (_mono_UiO-66) MOF.^107^

One of the key challenges in advancing adsorption technologies lies in the hesitation to report true volumetric adsorption capacities based on real – rather than single crystal – densities. This reluctance is influenced by several factors. Firstly, measuring density, with multiple definitions including skeleton, envelop and bulk, is inherently challenging, complicating the experimental process. Additionally, there is a common lack of awareness among researchers about the significance of volumetric data. However, understanding the importance of sol–gel processes in densifying MOFs and COFs sheds light on the significance of such measurements. By avoiding high mechanical pressures, these processes yield materials with exceptional volumetric adsorption performances. Indeed, while some MOFs may not be optimal with respect to mechanical stability, and cannot be shaped using mechanical pressure due to pore collapse, they can be effectively shaped and densified using a sol–gel method. For example, while HKUST-1 tablets prepared under 100 bar pressure shown a reduced BET area of 600 m^2^ g^−1^, robust HKUST-1 monoliths showed a gravimetric BET area of 1550 m^2^ g^−1^, much higher than *via* mechanical pressurization.^155^ Not only the gravimetric BET area is affected but the volumetric shows the impact of densification, with BET areas of 925 m^2^ cm^−3^ for the powder compared to 1651 m^2^ cm^−3^ for the monolith.^156^ In the case of ZIF-8, _mono_ZIF-8 showed a gravimetric BET area comparable to its powder equivalent (*ca.* 1400 m^2^ g^−1^).^131^ However, considering the density of the monolith *versus* that of the powder, the volumetric BET area of the monolith reached 1660 m^2^ cm^−3^*vs.* 485 m^2^ cm^−3^ for the non-densified powder. This metric not only serves as a favorable indicator of dense monolithic material performance but also offers a more relevant measure of gas adsorption potential in real-world applications. While volumetric capacity might seem inconsequential in laboratory settings, it holds significant value in large-scale industrial applications and not only in automotive. Higher volumetric capacities facilitate a reduction in the footprint of adsorption systems, ultimately lowering costs. Thus, despite the challenges associated with measuring density, recognizing the practical implications highlights the importance of reporting real volumetric adsorption capacities.

**Fig. 13:**
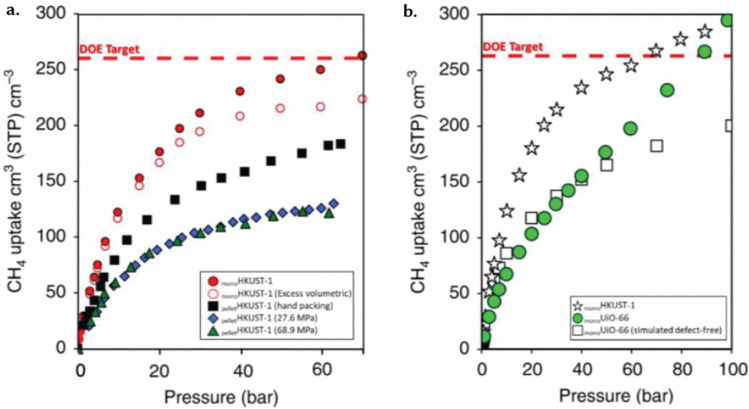
Monolithic MOFs exhibit volumetric BET areas that are three times higher compared to their powdered form. Additionally, they exhibit exceptional mechanical properties – surpassing the elastic modulus and hardness of single crystals. They also exhibit superior chemical stability. _mono_HKUST-1 displayed a remarkable methane volumetric adsorption of 259 cm^3^ (STP) cm^−3^ at 65 bar (a) the absolute volumetric methane adsorption isotherms at 298 K on monoliths and pellets of HKUST-1. _mono_UiO-66 achieved 296 cm^3^ cm^−3^ at 100 bar, a value comparable to that of _mono_HKUST-1 under high-pressure conditions. (b) Comparison of experimental isotherms for absolute volumetric CH_4_ uptake at 298 K in _mono_UiO-66 and _mono_HKUST-1. Reproduced under the terms of the CC-BY 4.0 licence from ref. 127 The U.S. DOE volumetric CH_4_ storage target of 263 cm^3^ (STP) cm^−3^ (65 bar) is indicated by the dashed red line.

The storage of natural gas, particularly through methane adsorption, has been a key focus in the development of high-performance porous materials. Among these, _mono_HKUST-1 and _mono_UiO-66 have demonstrated exceptional methane storage capacities, reaching 259 and 211 cm^3^ (STP) cm^−3^, respectively, at 65 bar and 25 °C (Fig. 13a and b).^18,107^ Notably, _mono_HKUST-1 became the first porous material to meet the U.S. Department of Energy (DOE) target for methane storage, marking a significant milestone in this field. By comparison, conventional HKUST-1 powders, which had one of the highest methane storage capacities among pristine MOFs, exhibited a significantly lower total methane storage capacity, reaching only 185 cm^3^ (STP) cm^−3^ g^−1^ L^−1^ at 65 bar and 298 K (Fig. 13a). This value was achieved through pelletization at 27.6 MPa as a structuring technique.^157^ However, advancements in material engineering have since led to the development of dense sol–gel monoliths, which have surpassed these storage capacities. More recently, in hydrogen storage, _mono_HKUST-1 demonstrated an impressive capacity of 46.0 g L^−1^ at 100 bar and 77 K.^156^ These results demonstrate the potential of monolithic porous materials in improving gas storage technologies and meeting energy targets.

In addition to densification, sol–gel, self-shaped monoliths typically display higher mechanical properties, surpassing the elastic modulus – up to more than twice – and hardness – up to 30% – compared to single crystals.^127^ Regarding the hydrochemical stability, _mono_ZIF-8 retained its crystalline structure and monolithic morphology after being submerged in boiling water for 7 days.^131^ This additional stability is an opportunity to use sol–gel monoliths in more demanding conditions. For example, we have studied three monolithic Zr-MOFs for water adsorption: _mono_UiO-66, _mono_UiO-66-NH_2_, and _mono_Zr-Fumarate.^158^ Looking at the best overall performer, _mono_Zr-Fumarate showed similar gravimetric BET area (854 m^2^ g^−1^) than the powder but far superior volumetric BET area, with 1063 and. 649 m^2^ cm^−3^ for the monolith and powder, respectively. Notably, the three monolithic materials showcased superior volumetric water uptake performance at 25 °C and 90% RH compared to their powdered counterparts, with 0.30, 0.50 and 0.31 g cm^−3^ for _mono_UiO-66, _mono_UiO-66-NH_2_, and _mono_Zr-Fumarate. Particularly noteworthy is the scale-up synthesis of the _mono_ZrMOFs to a multigram scale, facilitating the processing of 1 L of Zr-Fumarate colloid to yield 67 g of _mono_Zr-Fumarate. This scaled-up synthesis yields self-assembled monoliths while retaining all porosity and crystalline properties, signifying a notable development towards the practical industrial implementation of monolithic MOFs.^158^

**Fig. 14:**
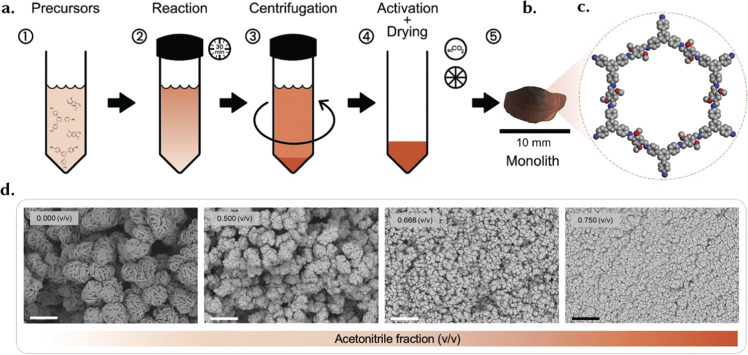
A self-shaped monolithic COF using TPB and DMTP as building blocks for gas separation. (a) Processing workflow for TPB-DMTP-COF monolith formation. (b) An optical image of TPB-DMTP-COF monolith. (c) Pore structure of TPB-DMTP-COF (C atoms are in grey, N atoms are in blue, and O atoms are in red; H atoms have been omitted for clarity). (d) SEM images of the TPB-DMTP-COF monolith synthesized with varying acetonitrile fractions (inset) – scale bar of 1 μm. Adapted (reprinted) under the terms of the CC-BY 4.0^154^ Copyright 2022, Elsevier.

On carbon capture, Fan *et al.* reported a γ-cyclodextrin-based monolithic MOF, _mono_γ-CD-MOF(K).^159^ This monolithic material demonstrated high performance in terms of volumetric CO_2_ uptake, achieving values of 44.04 and 36.68 cm^3^ (STP) cm^−3^ at 1 bar and 273 and 298 K, respectively, an improvement compared to the CO_2_ uptake reported values of the powdered counterpart: 37.23 and 30.65 cm^3^ (STP) cm^−3^ at 1 bar and 273 and 298 K, respectively. The selectivity of CO_2_ over N_2_ was of 36.5, and it displayed water stability after exposure to a 60% RH environment for 14 days. Breakthrough gas separation experiments using a 15/85 v/v CO_2_/N_2_ mixture—representative of post-combustion carbon capture—under both dry and 74% RH conditions showed that _mono_HKUST-1, _mono_UiO-66, and _mono_UiO-66-NH_2_ exhibited volumetric CO_2_ uptake values of 22.6, 16.0, and 20.0 cm^3^ cm^−3^, respectively.^160^ These values were significantly higher than their powdered counterparts, which reached only 12.4, 10.0, and 11.6 cm^3^ cm^−3^ in dry conditions.^160^ Even in humid environments, the monolithic materials maintained nearly double the CO_2_ uptake compared to their powdered forms. Similarly, for 50/50 v/v CO_2_/CH_4_ separation, relevant to natural gas purification, the _mono_HKUST-1, _mono_UiO-66, and _mono_UiO-66-NH_2_ monoliths exhibited superior volumetric adsorption performance, reaching 56.5, 42.0, and 36.2 cm^3^ cm^−3^, respectively. These values were significantly higher than those of their powdered analogues, which showed 30.8, 24.6, and 25.5 cm^3^ cm^−3^.^160^ The monoliths maintained consistent performance even after undergoing five adsorption/desorption cycles, highlighting their robustness and stability.^160^ Moving to COFs, we reported a self-shaped monolith using 1,3,5-tris(4-aminophenyl)benzene(TPB) and 2,5-dimethoxyterephthal-aldehyde (DMTP) as building blocks (Fig. 14).^154^ The COF monoliths were synthesized using a mixture of dioxane and acetonitrile. Using an acetonitrile volume fraction of 0.75 (v/v), the particle size was reduced to 40 nm, a size similar to what has been previously reported as necessary for achieving MOF monoliths (Fig. 14c).^154^ The gravimetric BET surface area of this material, 2125 m^2^ g^−1^, is slightly lower but comparable to the values reported for non-self-shaped materials of similar COFs. For example, the BET surface area of 2535 m^2^ g^−1^ was reported for TAPB-PDA-AG COF aerogels by Illán *et al.*^161^ However, in terms of volumetric BET area, the self-shaped monoliths surpass the aerogel, with 332 *vs.* 43 m^2^ (STP) cm^−3^. Dynamic breakthrough studies done with the obtained COF monoliths and using mixed gas feeds showed very similar separation performance for a 15% CO_2_ – 85% N_2_ mixture, and a noticeably sharper separation for the 50% CO_2_ – 50% CH_4_ mixture. For both cases, we observed an increase in CO_2_ capacity (13.4% and 8.6%, respectively).

Furthermore, monolithic synthesis provides a pathway for structuring composite materials as well. Within the subfamily of MOFs, ZIFs especially stand out for their zeolitic topologies. ZIF-8, in particular, is a flexible MOF^162^ extensively studied for its ability to grow around smaller molecules, essentially acting as a scaffold, and encapsulating guest molecules. Mehta *et al.* reported the synthesis of a composite with SnO_2_ NPs encapsulated using _mono_ZIF-8.^163^ To obtain the pristine MOF monolith, the reaction time was reduced to 15 minutes, maintaining a particle size of 60–80 nm. In contrast, for SnO_2_@_mono_ZIF-8, SnO_2_ NPs were added to the 2-methylimidazole solution before mixing. This method yielded particle sizes ranging between 100 and 150 nm, suggesting that ZIF-8 nucleated at a slower rate in the presence of SnO_2_ NPs. The composite exhibited moderate activity for the photocatalytic degradation of methylene blue (MB), achieving an average degradation of 41.5% with 53 μmol SnO_2_-NPs loading. Subsequently, the degradation increased to 97.6% with a catalytic loading of 225 μmol. However, aggregation of SnO_2_ NPs indicated their stabilization in the interparticle space rather than within the pores of the MOF, limiting their dispersion and loading capacity.^163^ Ye *et al.* encapsulated sulforhodamine 640 (SRh) within _mono_ZIF-8 – SRh, noted for its high photoluminescence quantum yield of around 63.6%, is an excellent candidate for laser gain mediums.^164^ The transparent ZIF-8 framework serves here as an effective scaffold for the dye, enhancing its optical performance. When excited with 532 nm laser pulses (the second harmonic of an Nd laser), SRh@ZIF-8 displayed a sharp emission peak near 620 nm at an energy density of 31 μJ cm^−2^. This represented the lowest threshold reported for SRh-doped polymers and MOF-based gain media at the time, surpassing SRh-doped polymers, which typically require 53 to 95 μJ cm^−2^, and other MOF-based media with thresholds ranging from 41 to 7.5 × 10^6^ μJ cm^−2^. The observed narrowing of the emission band with increasing energy density indicated amplified spontaneous emission (ASE), reflecting the high optical quality of SRh@ZIF-8. With dimensions of approximately 6 × 3 × 1 mm^3^, the composite is up to 10 000 times larger than conventional MOF crystals used in laser tests, demonstrating its potential for large-scale applications in laser devices and photonic technologies.

Tian *et al.* successfully encapsulated gold nanoparticles (Au NPs) within _mono_ZIF-67 by reducing the reaction time to 30 minutes and conducting the reaction at 0 °C, resulting in Au NP sizes of approximately 140 nm, which is significantly smaller than the 260 nm particles obtained from earlier room temperature methods.^165^ SEM imaging confirmed the uniform distribution and effective encapsulation of Au NPs within the ZIF-67 framework. The synthesis of _mono_ZIF-67 at 0 °C using a sol–gel method led to a structure with a higher volumetric BET area and CO_2_ adsorption capacity compared to ZIF-67 powder, which in turn resulted in a 90% increase in the volumetric CO production rate. 8 mLAu@ZIF-67, the best performer, exhibited a volumetric CO production rate 1.5 times greater than _mono_ZIF-67 and 3 times greater than ZIF-67 powder, reflecting a beneficial combination of high gravimetric yield and bulk density. Mechanical testing showed that 8 mLAu@ZIF-67 had an elastic modulus of 3.53 ± 0.25 GPa and hardness of 0.316 ± 0.040 GPa, which are similar to _mono_ZIF-67's values of 3.47 ± 0.15 GPa and 0.341 ± 0.027 GPa, indicating that the presence of Au NPs had minimal effect on mechanical stability. The successful synthesis of Au@ZIF-67 demonstrated improved CO_2_ photoreduction performance, highlighting its potential for applications requiring both high performance and durability.^165^

#### 4.1.2. Dip coating

Dip-coating is a simple and widely adopted solution-based technique for depositing thin films and coatings on various substrates, ranging from metallics and ceramics to polymer films and fibers. The process involves immersing the substrate into a solution of the coating material, ensuring complete infiltration, followed by withdrawing the substrate from the solution. Subsequently, the wet coating sediments are evaporated to achieve dryness.^166^ Mori and colleagues developed a strategy of submerging Cu-based reactors into an acidic solution containing an organic linker – facilitating the *in situ* synthesis of HKUST-1 crystals on the reactor surface.^167^ The formation of the MOF layer not only served as a catalyst for the deposition of active metal NPs but also led to the formation of carbonaceous layers through pyrolysis under inert conditions. This facilitated further functionalization with organic modifiers, *e.g.*, *p*-phenylenediamine, and metal NPs, *e.g.*, catalytically active Pd NPs, resulting in 3D-printed reactors that showed promise for catalytic hydrogen production from liquid-phase hydrogen carriers. They then employed laser powder bed fusion (LPBF) to craft Cu-based reactors tailored for catalytic functions. LPBF, an additive manufacturing technique utilizing a high-power laser to selectively meld metallic powder layers, allowed for the creation of intricate three-dimensional structures with exceptional precision. By submerging Cu-based reactors into an acidic solution containing an organic linker, they facilitated the on-site synthesis of HKUST-1 crystals directly onto the reactor surface, enabling the fabrication of reactors with customized geometries and internal architectures, thereby enhancing their efficacy for catalytic applications. The resulting internal structure significantly influenced catalytic activity, as evidenced by the observed inverse correlation between pressure loss magnitude and pore size. Through LPBF-based adjustments in cell density and internal geometry, they could fine-tune reactors for specific catalytic processes, such as hydrogen generation from liquid-phase carriers.^167^ Gkaniatsou *et al.* employed dip coating to deposit MOFs onto dehumidification heat exchangers using silicon as the binder, showcasing successful implementations.^168^ They highlighted the use of water-stable Al-MOFs in adsorption cooling, synthesized through eco-friendly processes. MIL-160, CAU-10, Al-Fum, and CAU-23 exhibited potential, achieving thermal efficiencies above 0.6 and specific cooling powers exceeding 1 kW kg^−1^. When powered by solar thermal energy, Al-MOFs maintained stable energy conversion efficiencies despite varying conditions. This approach ensured stable adherence, even through multiple temperature swing cycles. Dip coating ensured uniform coatings for optimal performance, although challenges with coating homogeneity arose at scale. The Al-MOF adsorption chiller showed promise for sustainable cooling, with future research aiming to enhance efficiencies and reduce costs for broader adoption alongside increasing renewable energy integration.^168^

In another work, Sarango *et al.* developed a dip-coating method for creating thin film nanocomposite (TFN) membranes with precise MOF (ZIF-8 and ZIF-67) nanoparticle arrangements.^169^ This method reduces clumping and conserves reactants. For example, using ZIF-8 particles of 70 ± 10 nm provided better uniformity and coverage than ZIF-67 particles of 240 ± 40 nm, resulting in improved performance. The dip-coating process ensured MOF particle deposition without loss during interfacial polymerization. TFN ZIF-8 membranes showed increased methanol permeance (up to 8.7 L m^2^ h^−1^ bar^−1^; a 150% rise compared to thin film composite membranes) while maintaining high rejection rates, indicating effective substance blocking and selective permeation.^169^ COF membranes can also be fabricated through a dip-coating process. For example, Tsuru and coworkers created COF-1 nanosheets obtained by sonication of bulk COF-1 materials.^170^ These nanosheets were then deposited onto the external surface of SiO_2_–ZrO_2_-modified α-Al_2_O_3_ supports using a drop-coating method, followed by drying at room temperature. The COF-1 membranes, about 100 nm thick, demonstrated high hydrogen gas permeability, reaching 17 mol m^−2^ Pa^−1^ s^−1^ at 25 °C due to the perforations in the nanosheets. N_2_ adsorption isotherms showed that pristine COF-1 had a pore size of approximately 0.6 nm, with uniform 1.5 nm perforations in staggered nanosheet stacking. Moreover, the COF-1 nanosheets exhibited good thermal stability due to robust covalent bonds within the structure.^170^

Apart from these top-down strategies, some reports about COF membranes are based on a bottom-up approach. For example, Park *et al.*^171^ deposited nine different COFs built using the same aldehyde precursor, 1,3,5-triphloroglucinol, on the surface of Zn electrodes by immersing a Zn foil into the COF precursor's solution. Then, they used an imine condensation reaction on the Zn surface to create uniform COF films of 30 × 12 cm^2^ on both planar and curvilinear supports. The COF films showed strong affinity to Zn^2+^ ions due to favorable interactions with the electron-rich ketone and imine functional groups in the COFs, allowing for efficient mass and charge transport, and suppressing large Zn dendrites. The COF films did not show any noticeable cracks and deterioration after 200 folding/recovery cycles.^171^

Dip coating has also been used in sensing applications. Demessence *et al.*^172^ synthesized stable, 22 ± 5 nm monodisperse MIL-101(Cr) NPs using a green microwave method at 200 °C for 1 minute.^172^ The NPs, when dispersed in ethanol, can be stored for up to 2 months without structural changes. The NPs exhibit a Langmuir surface area of 4200 ± 80 m^2^ g^−1^, which is consistent with the bulk material. However, it is important to note that while the Langmuir model provides an estimation of surface area, it is not the most appropriate method for characterizing porous materials due to its inherent assumptions regarding monolayer adsorption. Thin films of MIL-101(Cr) deposited *via* dip-coating produce uniform films, the thickness of which depends on the concentration of the NP suspension. The films demonstrate two-step water adsorption, corresponding to the two mesoporous cage sizes in MIL-101(Cr), with porosity reaching 78%, encompassing both the NPs and inter-NP space. Mechanical testing reveals the rigid nature of the NPs (Young's modulus ≈ 17 ± 10 GPa), while the overall film exhibits lower rigidity (≈ 40 ± 10 MPa) due to its high porosity. Adsorption isotherms with alcohols showed reversible adsorption, indicating potential for selective adsorption properties.^172^ Overall, immersion time, temperature, concentration, and viscosity of the targeted suspension, together with the substrate surface properties, play significant roles in determining the thickness, distribution, and morphology of the thin films. Control over these parameters is essential to optimize their performance. Given these advantages, however, dip-coating techniques face challenges in terms of scalability, reproducibility, and parameter optimization.

#### 4.1.3. Deposition techniques

Vapor deposition is a broad category of thin film deposition methods that involve vaporizing a source material into a gaseous state, to then condense onto a substrate. Vapor deposition can be classified into physical and chemical vapor deposition (PVD and CVD, respectively). PVD involves transforming a solid material into vapor through a physical process and, therefore, occurs typically in a vacuum environment to minimize gas interactions and improve film quality. However, since this method requires elevated temperatures, it is challenging for MOFs and other reticular materials due to their limited thermal stability, with limited reports.^173^ On the other hand, CVD involves controlled chemical reactions of gaseous precursor molecules on a substrate surface, resulting in the formation of a solid film layer with controlled thickness. This is a more versatile processing technique due to its scalability and, in certain cases, its ability to occur without necessitating a vacuum environment.^174^

**Fig. 15:**
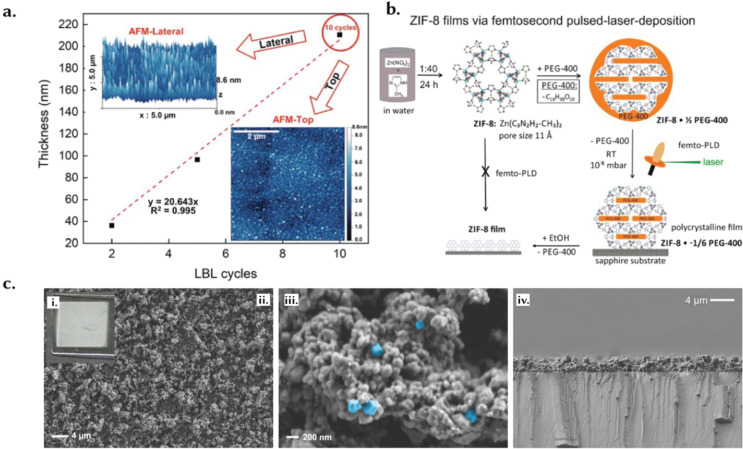
(a) Using a layer-by-layer growth approach, H_3_BTC was sequentially deposited on a SiO_2_/Si(100) substrate using CVD, and Cu using PVD. The graph shows the thickness of the resulting HKUST-1 thin film as the cycles of the layer-by-layer growth progress. Top and lateral views of the HKUST-1 thin film after 10 cycles as measured by AFM are inset. Reprinted with permission from ref. 175 Copyright 2019 Royal Society of Chemistry. (b) Femto-PLD technique for the fabrication of ZIF-8 thin films. A schematic illustration of the steps used for the fabrication of the thin films. Reprinted with permission from ref. 176 Copyright 2017 American Chemical Society. (c) Visualising the thin films: (i) optical image of the thin film on a sapphire substrate, (ii) SEM image of the thin film (top view, scale bar: 4 μm), (iii) SEM image of the thin film (top view, scale bar: 200 nm) – crystals showing ZIF-8 morphology have been false-coloured as blue, (iv) SEM image of the thin film (cross-sectional view, scale bar: 4 μm). Reprinted with permission from ref. 176 Copyright 2017 American Chemical Society.

Despite the limitations of PVD on reticular, porous materials, PVD can also be combined with CVD. For example, Han *et al.* prepared HKUST-1 films using this approach (Fig. 15a).^175^ They first created a 1 nm film directly on a SiO_2_/Si(100) substrate using PVD, which served as the support for the subsequent deposition of H_3_BTC *via* CVD at 200 °C. Glancing-angle X-ray diffraction (GAXRD) showed two distinct planes at (220) and (222), indicating the formation of highly oriented HKUST-1 thin film. In addition, Fischer *et al.* developed a femtosecond pulsed-laser deposition (femto-PLD) technique for fabricating ZIF-8 thin films (Fig. 15b).^176^ This approach extends the available film fabrication techniques for MOFs, effectively sidestepping challenges associated with decomposition or amorphization. In this study, they used PEG-400 as a stabilizing agent for the deposition of ZIF-8, with the PEG being removed by washing with ethanol after the formation of the ZIF-8 films. This methodology yielded mesoporous ZIF-8 films constituted by nanoscale ZIF-8 crystals, as confirmed by SEM (Fig. 15c) and 77 K N_2_ isotherms.

Mondloch *et al.*^177^ reported the first application of ALD – a variant of CVD based on sequential, self-limiting reactions that facilitate thickness control at the angstrom level^176^ – for incorporating single atom sites (Zn and Al) inside the structure of NU-1000. This approach, termed ‘atomic layer deposition in MOFs’ (AIM), allowed to enhance the catalytic performance of NU-1000 in Knoevenagel condensation reactions. NU-1000, synthesized *via* solvothermal reactions, demonstrated thermal stability up to 500 °C, mesoporous channels, and strategically positioned –OH groups required for the metalation. Diethylzinc (ZnEt_2_) and trimethylaluminum (AlMe_3_) were used as ALD precursors to achieve metalation, giving an average of 0.5 Zn (Zn-AIM) and 1.4 Al (Al-AIM) atoms per Zr atom. NU-1000 retained its crystallinity during the process, while the BET area decreased from 2230 m^2^ g^−1^ for pristine NU-1000 to 1580 and 1160 m^2^ g^−1^ for the Zn- and Al-doped NU-1000, respectively, due to the extra metals and the space taken from the porosity.^175^ Following on ALD, Stassen *et al.* showed the preparation of ZIF-8 films through a two-step ‘MOF-CVD’ method: a metal oxide deposition step and a vapor–solid reaction step.^178^ The initial deposition involved creating a ZnO layer *via* ALD followed by the introduction of 2-methylimidazole organic linker vapor into the reaction system *via* CVD. Notably, the solvent-free nature of the MOF-CVD process allowed for lift-off patterning and the fabrication of MOF films on delicate substrates. In a separate study, Liu and coworkers used vapor-induced conversion within CVD to synthesize a series of large-area COF films featuring –C=N– linkages, termed PyTTA-TPA, PyTTA-BPyDCA, and PyTTA-BPDA, with controllable thicknesses.^179^ Among them, the carrier mobility in a 30-nm-thick PyTTA-TPA COF film reached 1.89 × 10^−3^ cm^2^ V^−1^ s^−1^, significantly higher than that of PyTTA precursors. The authors attributed this enhancement to charge transport through the COF lattice. Additionally, the film demonstrated notable electrocatalytic activity for the hydrogen evolution reaction (HER), outperforming metal-free COFs and even certain metallic catalysts.

#### 4.1.4. Spray drying

Spray-drying involves the rapid atomization of a solution or suspension of multi-components of the desired materials into an aerosol droplet, followed by rapid solvent evaporation under hot air at a certain temperature and pressure, leading to the formation of a solid powder, normally with a spherical morphology.^180^ During this process, several key parameters need to be controlled and optimized, including the feed rate at which the precursors are injected, the flow rate at which the atomization occurs, and the temperature of the gas that is used to dry the droplets formed. These parameters impact both at a process level (*e.g.*, throughput, cost, and quality) and the material itself (*e.g.*, size, shape, and morphology). Spray drying offers continuous manufacturing and maximizes throughput, ensuring consistency and efficiency throughout production.^180^

**Fig. 16:**
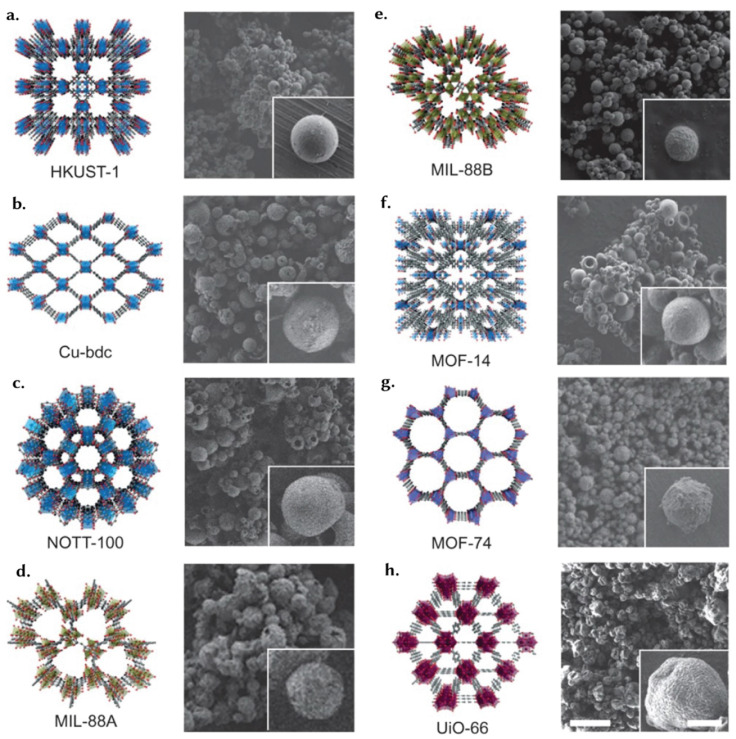
MOFs synthesized by the spray-drying approach (left: crystal structure, right: SEM images of the MOF superstructures and discrete nano-MOF crystals (inset)). (a) HKUST-1 (b) Cu-bdc. (c) NOTT-100. (d) MIL-88A. (e) MIL-88B. (f) MOF-14. (g) Zn-MOF-74. (h) UiO-66. Adapted with permission from ref. 181. Copyright 2013 Springer Nature.

Pioneering work in this area has been carried out by Maspoch and co-workers. First, Carné-Sánchez *et al.* introduced spray-drying for MOF synthesis (Fig. 16).^181^ They focused on the formation and subsequent drying of droplets containing the metal salt and the corresponding organic linker. Upon the rapid evaporation of the droplets, MOF nanocrystals were formed at the air–liquid interface. These nanocrystals merge to form hollow superstructures, whose size can be precisely controlled based on the type of MOF and the synthesis conditions, ranging from tens to hundreds of nanometers. The first example was using HKUST-1 by directly injecting a solution of Cu(NO_3_)_2_·2.5H_2_O and H_3_BTC in mixed solvents of DMF, EtOH, and H_2_O (Fig. 16a). This approach was later expanded to other MOFs, including Cu-bdc, NOTT-100, MIL-88A, MOF-14, MOF-74, and UiO-66 (Fig. 16b–h).^181^ In addition to creating hollow structures, spray drying can generate dense ones. In this regard, Mitsuka *et al.* developed a two-step synthesis process for MOFs, focusing on those with high-nuclearity secondary building units—clusters containing multiple metal atoms that enhance stability and connectivity.^182^ The UiO-66 family exemplifies this, featuring Zr_6_-based clusters that provide exceptional structural robustness. First, MOF seeds were generated by heating mixed precursors at a specific temperature. The seed suspension was spray-dried to promote crystal growth, resulting in spherical UiO-66 superstructures with diameters ranging from half a micron to a few microns, while the primary MOF particles typically remained below 100 nm in size. The process also allowed for control over particle morphology while facilitating MOF production.^181^ Garzon-Tovar *et al.* also developed a synthesis method for high-nuclearity MOFs by combining continuous flow and spray-drying synthesis.^183^ This dual approach – comparable to the work of Mitsuka *et al.* – was designed to produce spherical microbeads of MOFs, including UiO-66. Here, the continuous flow reactor ensured MOF nucleation and avoided the formation of amorphous products, which was an issue in earlier studies.^181,182^ The optimized process parameters included a feed rate of 2.4 mL min^−1^, a flow rate of 336 mL min^−1^, and an inlet temperature of 180 °C – these three parameters, and their impact on the quality of the materials through their BET areas, were largely explored. These conditions allowed complete solvent evaporation and resulted in spherical microbeads with an average diameter of 4.3 ± 2.6 mm, composed of nanoparticle aggregates. The resulting UiO-66 beads exhibited a BET area of 1106 m^2^ g^−1^, similar to the one obtained using conventional methods. The method was also expanded to other high-nuclearity MOFs, including Fe-BTC/MIL-100 and [Ni_8_(OH)_4_(H_2_O)_2_(L)_6_]_*n*_. Fe–BTC/MIL-100 yielded 78% with a BET area of 1039 m^2^ g^−1^, while [Ni_8_(OH)_4_(H_2_O)_2_(L)_6_]_*n*_ showed a yield of 60% and a BET area of 377 m^2^ g^−1^. Additionally, the method demonstrated flexibility in producing multivariate (MTV) MOFs, such as UiO-66, using different organic linkers.^183^

Camur *et al.* advanced the combined continuous-flow and spray-drying method on UiO-66-NH_2_ and explored the effect of acetic acid as a modulator and using water as a solvent.^184^ At 14% acetic acid, the microbeads exhibited a BET surface area of 840 m^2^ g^−1^, which increased to 1036 m^2^ g^−1^ at 56%. However, at 70% acetic acid, the BET area decreased to 655 m^2^ g^−1^ due to competition between the modulator and ligand, affecting crystallinity. The optimal concentration of 30% acetic acid resulted in microbeads with a particle size distribution of 4–10 μm, a BET surface area of 1261 m^2^ g^−1^, and a water uptake of 0.57 g g^−1^ at 0.2 *P*/*P*_0_. They expanded the method to Zr-fumarate, obtaining beads with a BET area of 664 m^2^ g^−1^ at 30% acetic acid.^185^ Though slightly lower than hydrothermal methods, the spray-drying technique demonstrated scalability, ease of use, and environmental benefits, emphasizing the importance of modulator concentration in tuning MOF properties.^184^ Boix *et al.* improved the integration of inorganic nanoparticles (iNPs) into 1.5 μm UiO-66 microbeads using a flow reactor at 115 °C.^186^ The UiO-66 and CeO_2_@UiO-66 microbeads, along with their thiol-functionalized derivatives (UiO-66-(SH)_2_ and CeO_2_@UiO-66-(SH)_2_), were synthesized using a continuous-flow spray-drying technique, forming spherical microbeads (average size: 1.5 ± 1.0 μm) composed of UiO-66 nanocrystals and CeO_2_ nanoparticles. These microbeads showed high porosity, with BET surface areas of 945 m^2^ g^−1^ for UiO-66, 597 m^2^ g^−1^ for UiO-66-(SH)_2_, 747 m^2^ g^−1^ for CeO_2_@UiO-66, and 539 m^2^ g^−1^ for CeO_2_@UiO-66-(SH)_2_. They effectively removed heavy metals such as As(iii and v), Cd(ii), Cr(iii and vi), Cu(ii), Pb(ii), and Hg(ii) from a solution with 100 ppb concentration (for each metal ion precursor), with removal efficiencies of 99% for Pb(ii) and Cu(ii), 98% for Hg(ii), 93% for Cr(iii and vi), and 56% for As(iii and v). Thiol-functionalization enhanced adsorption capacities for Pb(ii), Cu(ii), and Cr(iii and vi), while CeO_2_ improved the removal of As(iii) and Cr(iv). The microbeads exhibited stability during adsorption, with no detectable release of Zr(iv) or Ce(iv) ions and retained crystallinity after metal adsorption. In a continuous-flow column, CeO_2_@UiO-66-(SH)_2_ microbeads removed 99% of Pb(ii) and Hg(ii), 85% of Cd(ii), 84% of Cr(iii and vi), and 69% of As(iii and v) at a flow rate of 1.3 mL min^−1^, with a breakthrough time of 231 minutes (300.6 mL) and a maximum Cr(iii) loading capacity of 82.7 mg g^−1^. The microbeads were regenerated easily with an acidic treatment, achieving desorption rates exceeding 96%. In real river-water samples from the Buringanga, Bone, and Sarno Rivers, the microbeads reduced metal concentrations below WHO limits, demonstrating their effectiveness in real-world water purification. A magnetic version of CeO_2_@UiO-66-(SH)_2_ microbeads, incorporating Fe_3_O_4_ nanoparticles, allowed easy recovery from water using a magnet, maintaining their metal-adsorption capacity. The microbeads maintained integrity over three cycles, achieving a maximum capacity of 82.7 mg (Cr(iii)) g^−1^, and the Fe_3_O_4_-enhanced version enabled efficient recovery without compromising performance, further broadening their potential applications in water treatment. Continuing their work, they incorporated CeO_2_-doped UiO-66 microbeads into porous polyethersulfone (PES) structures *via* spray-drying,^187^ achieving CeO_2_ encapsulation levels of 4.0% and 3.3%, with yields of 93% and 87%. Nitrogen adsorption confirmed a BET area of 945 m^2^ g^−1^ for UiO-66. In continuous-flow tests, 10 mg of microbeads removed over 99% of Pb(ii), Hg(ii), and Cu(ii) from 30 mL of water. Tests in river water from Bangladesh, Indonesia, and Italy showed over 98% removal of Cd(ii), Cu(ii), and Pb(ii), with Cr(vi) and As(iii) reduced to safe levels. The magnetically functionalized CeO_2_/Fe_3_O_4_@UiO-66-(SH)_2_ microbeads demonstrated excellent performance and easy recovery, making them suitable for large-scale water purification applications.

With some modifications, spray drying can be also translated to COFs. The challenge here is that COFs are typically formed under thermodynamic control while the spray-drying approach hinges on kinetic control – *i.e.* rapid product formation – which may not necessarily be thermodynamically stable and may result in different phases, including non-porous ones.^180^ Garzon-Tovar *et al.*^188^ introduced a method combining spray-drying with dynamic covalent chemistry to synthesize zero-dimensional spherical COF superstructures from imine-based nanocrystals. This two-step approach first forms amorphous polymer spheres *via* spray-drying, which are then crystallized into COFs like COF-TAPB-BTCA, COF-LZU1, and COF-TAPB-PDA. The resulting microspherical superstructures retain their size and shape after crystallization, with COF-TAPB-BTCA showing a BET surface area of 911 m^2^ g^−1^, COF-LZU1 at 319 m^2^ g^−1^, and COF-TAPB-PDA at 1162 m^2^ g^−1^. Furthermore, the method allows for the integration of functional materials, creating composites such as Rose-bengal@COF-TAPB-BTCA with uniform dye distribution and slow release, and Fe_3_O_4_@COF-TAPB-BTCA composites that exhibited magnetic properties with 2.8% Fe_3_O_4_ content and easy magnet retrieval. This approach expands the potential of COFs for applications requiring structured materials with enhanced properties.

### 4.2. Post-synthetic shaping and densification

While Section 3 describes the main differences and characteristics of conformed and densified bodies, the current section reviews the methods used to prepare them. Post-synthetic shaping provides an important degree of flexibility in controlling the final shape and body density by enabling control over compression conditions.^117^ To ensure optimal mechanical properties, these bodies require the introduction of binders and plasticizers.^189^ The primary function of the binder is to glue the MOF powder particles together, while the plasticizer serves to increase the plasticity of the binder, making it less brittle. Binder materials can be either inorganic, such as Al_2_O_3_ or silica, or organic, such as mono/polysaccharides (*e.g.*, cellulose) or polyvinyl alcohol (PVA); plasticizers include glycerol, propylene glycol, or triethyl citrate. Proper selection of the binder is crucial to the properties and performance of the resulting bodies to withstand crushing by attrition and heavy weight loads. Considerations such as surface tension and viscosity of the solution, as well as binder interactions with powder particles, are essential.^190^ These considerations help strike a balance between preserving the desired material properties in terms of pore volume, density, and adsorption capacity and achieving the necessary mechanical properties and thermal conductivity in the final product.

**Fig. 17:**
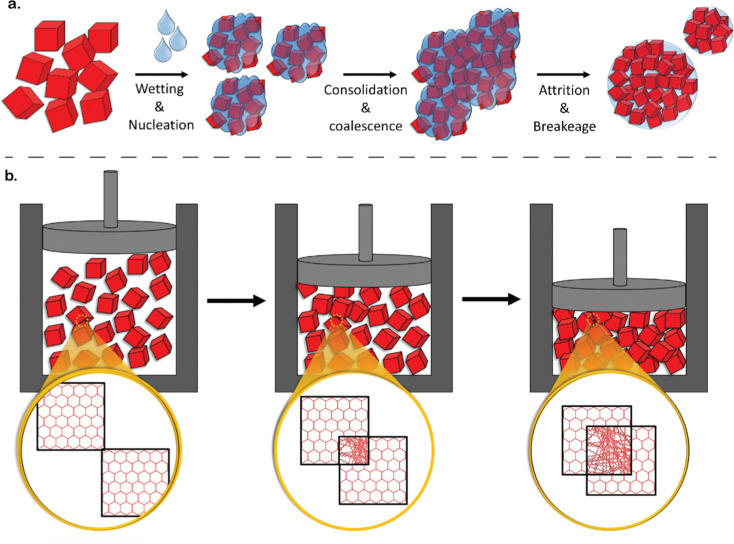
(a) The three steps for wet pelletization: (i) wetting and nucleation – when a volatile solvent (and if needed, a binder), is added and the mixture and the particles begin to aggregate, (ii) consolidation and coalescence – where the particles keep aggregating until particle size reaches its maximum, and (iii) attrition and breakage – where forces applied break and shape the material resulting in the final granules. (b) Partial amorphization of materials upon pelletization with pressure – leading to a reduction in the porosity of the material. As a consequence, depending on the application, values for these pelletization parameters need to be optimized.

Post-synthetic shaping can work differently when dealing with wet or dry samples. Solvents are often used to enhance binding between powder particles and binders, being removed during the drying, but they can also help to avoid pore collapse during the process.^191^ Wet processes takes place in three steps (Fig. 17a): (i) wetting and nucleation, when a volatile solvent and a binder are added and the mixture and the particles begin to aggregate; (ii) consolidation and coalescence, where the material starts aggregating until they reach a maximum size; and (iii) attrition and breakage, where the forces applied break and shape the material resulting in the final granules. In the cases where the material is sensitive to either the solvent or the heat required for drying – which can be the case in reticular materials – an alternative dry process that relies solely on mechanical compression may be employed.^190^ Overall, one of the main drawbacks of this shaping process is the need for a precise control over the total pressure applied, the increase rate, and the dwell time in the mold during the pelletization. The process will allow for the increase in the density of the material and, therefore, an improvement in its mechanical properties (*i.e.* higher attrition or breakage resistance) and volumetric adsorption properties. However, as explained in Section 3, the increase in the density can result in a gravimetric reduction of the porosity due to pore collapse due to mechanical compression; alternatively, binder addition can result in pore blocking.^131^ It is important to note that, beyond certain mechanical pressures, which depend on each specific MOF, the crystalline structure transforms into an amorphous phase and the collapse of the porosity (Fig. 17b). Here, it is important to remember that the mechanical properties of MOFs^36^ and other reticular materials^154^ depend first on topology and then on pore volume, pore size, and density *e.g.*, the smaller the cluster connectivity and the larger the pore volume and size, the lower the mechanical properties. Post-synthetic shaping and densification processes can be classified broadly into (i) tableting, (ii) extrusion, (iii) spherenoization, (iv) 3D printing, (v) phase inversion and hydrogelation, and (vi) glass formation.

#### 4.2.1. Tableting

Tableting is a process where porous materials are shaped into solid forms (*e.g.*, pellets and granules) by compressing powder particles in a mold under high mechanical pressure, often resulting in a denser structure while maintaining the material's chemical integrity and porosity. This method is commonly used in the production of tablets for pharmaceutical and material applications. Ardelean *et al.* compressed MIL-101 MOF powder into pellets, achieving a peak bulk density of 1.34 g cm^−3^ at 120 MPa, where the transition to an amorphous phase starts.^192^ However, at intermediate densities up to 0.47 g cm^−3^ (30–50 MPa), the crystal structure remained intact, and pellets within the 0.45 to 0.47 g cm^−3^ range exhibited a hydrogen storage capacity of 40 g L^−1^ at 196 °C and 8 MPa. At 77.3 K, hydrogen adsorption isotherms showed reduced surface area and micropore volume as density approached crystal density, while XRD indicated no significant structural changes until then. IR spectra revealed pressure-induced shifts in carboxylate and phenylene frequencies, paralleling findings in MOF-5 and MOF-177, which transitioned to amorphous phases at higher densities.^192^ Oh *et al.*'s research measured the hydrogen uptake capacity of MIL-101 in powder and pellet forms, showing that at 20 K, pellets can absorb up to 9.6 wt% and 42 g L^−1^ of hydrogen.^193^ This cryo-adsorption method offers a wider temperature range for storage without boiloff, making it ideal for industrial use. The hydrogen capacity at 20 K is nearly double that at 77 K due to pore condensation in MIL-101's cavities. The study suggests that combining cryo-adsorption with liquefaction at 20 K can reduce boiloff and extend the storage range to 37 K, with MIL-101 sorbents preventing rapid hydrogen expansion and improving system performance.^193^ Then, Blanita *et al.* developed hexagonal prism-shaped MIL-101 monoliths for hydrogen adsorption, achieving envelope densities up to 0.467 g cm^−3^ and good mechanical stability.^194^ At 77 K, excess hydrogen adsorption (*N*_ex_) decreased from 5.69% H_2_ to 4.54% H_2_ for pellets with a density of 0.467 g cm^−3^, and dropped to 2.4% H_2_ at 159 K. At 77 K and 150 bar, the total volumetric capacity was 46.5 g L^−1^, with a working capacity of 45 g L^−1^ after discharge at 159 K and 5 bar. MIL-101 pellets (0.4 g cm^−3^) stored 6.9 kg H_2_ at 100 bar and 7.9 kg H_2_ at 150 bar, corresponding to volumetric capacities of 36.4 g L^−1^ (6.2% H_2_) at 100 bar and 41.1 g L^−1^ (7.0% H_2_) at 150 bar. MIL-101(Cr) remained stable in air for 8 months, withstood temperatures up to 220 °C, and supported over 1500 cycles, making it suitable for low-purity hydrogen refuelling.

Tableting and pelletization can be applied beyond hydrogen storage. Permyakova *et al.* used MIL-127(Fe), MIL-125(Ti)-NH_2_, MIL-100(Fe), and MIL-160(Al) for water adsorption in heat storage.^195^ For example, they evaluated MIL-160(Al) granules in a pilot-scale reactor. Through a wet granulation process, the MOF powder was mixed with 10 wt% silica sol as a binder, and then shaped into spherical granules using a rolling machine. After drying at 100 °C for 12 hours, they obtained spherical macrostructures ranging from 0.5 to 1.8 mm in size. The BET area dropped from 1150 to 1000 m^2^ g^−1^ only, likely due to partial pore blockage caused by the binder. Despite these changes, MIL-160(Al) granules maintained good cycling loading lifts over 10 adsorption/desorption cycles, with water working capacities of 0.36 and 0.32 g_water_ g_adsorbent_^−1^ for powder and granules, respectively, measured between 30 °C for adsorption and 80 °C for desorption, at 1.25 kPa – corresponding to an energy capacity of 305 W h kg^−1^ under mild desorption conditions. Similarly, Kim *et al.* used wet granulation for MIL-100(Fe) and a silica sol binder for SF_6_/N_2_ separation, obtaining granules with sizes ranging 1.18–1.70 mm.^196^ The granulation process resulted in a slight reduction in BET area from 1772 m^2^ g^−1^ (powder) to 1619 m^2^ g^−1^. This is an 8.6% decrease in BET area, lower than the 33.6% reduction reported in pressed granules. In turn, the bulk density increased from 331 to 498 g L^−1^, enhancing the volumetric adsorption capacity. At the end of the day, the SF_6_ adsorption capacity of the granules (1.658 mmol g^−1^) was similar to the powder (1.673 mmol g^−1^), maintaining structural stability after high-temperature exposure and five adsorption/desorption cycles. Breakthrough experiments with 10 vol% SF_6_/N_2_ mixtures showed that MIL-100(Fe) granules had an SF_6_ breakthrough time that increased linearly with pressure. Although Zeolite 13X performed better at lower pressures, MIL-100(Fe) demonstrated improved performance at higher pressures, with faster regeneration (20 minutes *vs.* 250 minutes for Zeolite 13X) and consistent performance across five cycles, while Zeolite 13X experienced some performance decline due to difficult desorption. In another study, Martins *et al.* used a method to create MIL-100(Fe) granules by mixing the MOF powder with 10% silica binder and spraying water and ethanol, resulting in semi-spherical granules of 1.0–3.0 mm diameter, with a micropore volume of 0.58 cm^3^ g^−1^ and a BET area of 1568 m^2^ g^−1^.^197^ Using pressure swing adsorption (PSA), they achieved 99.5% ethane purity (86.7% recovery) and 99.4% propane purity (97.0% recovery) in 30/70 ethane/propane mixtures, and 100% ethylene purity and 94.7% propane purity (100% recovery) for 30/70 ethylene/propane mixture.^197^

When using pelletization to structure porous materials, several factors must be considered. One key issue is the mechanical properties of highly porous materials. While pelletization can increase volumetric capacities for adsorption, it can also lead to a 15–20% decrease in gravimetric capacities compared to the powder form. This reduction is primarily due to the loss of capacity from the addition of binders.^189^ While similar developments and densification techniques to the ones reported here have been widely used for materials such as zeolites, especially in applications such as chemical separation and conversion,^198^ the application of these techniques to newer classes of reticular porous materials has not yet been thoroughly investigated. Given the open nature of their porosities, these emerging classes of porous materials present unique challenges, and further research may be needed to adapt and optimize densification methods for their specific properties and applications.

#### 4.2.2. Extrusion

**Fig. 18:**
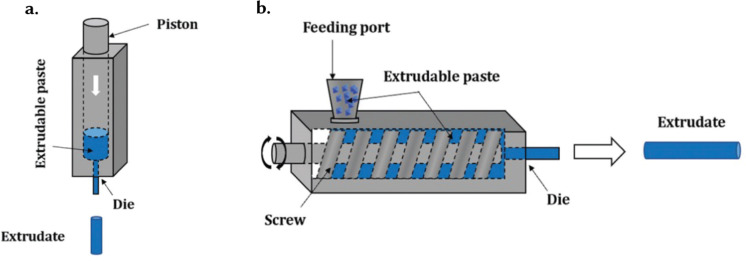
A schematic depiction of the extrusion process. A standard extrusion setup typically comprises a feeder module that operates *via* volumetric or gravimetric methods, and a barrel housing either piston screws, a screw or two screws. (a) A piston extrusion setup. (b) A screw extrusion setup. Reprinted with permission from ref. 119 Copyright 2021 Royal Society of Chemistry.

Extrusion is a well-established technique widely utilized to shape materials. Extrusion broadly involves the movement of material through confined spaces, typically facilitated by a piston or sets of screws. Standard extrusion setups comprise a feeder module, which operates through either volumetric or gravimetric methods, and a barrel housing either a piston screw (Fig. 18a), or two screws (Fig. 18b). In piston extrusion, hydraulic or pneumatic pressure is applied to materials within a die, enabling precise shaping of complex forms. On the other hand, in screw extrusion, the screw(s) convey the material through the interior of the barrel, subjecting it to shearing forces, before its eventual exit from the barrel. At the exit point, a die is used to shape the material according to the desired application. It is worth noting that similar setups are employed in mechanochemistry approaches, which are solvent-free strategies for the large-scale synthesis of materials.^199^ While the mechanochemistry approach to synthesis should be theoretically classified under *in situ* shaping techniques, it is discussed here.

In the case of MOFs, the extrusion process typically involves a combination of MOF powder and, like in pelletization, a binder, and a plasticizer. Extrusion is also typically performed in the presence of a solvent, following stages that include pressurization and final shaping.^200^ While in classical porous materials such as zeolites, binders can be subsequently removed through heat treatment processes (typically in the range of 300 to 1000 °C)^201,202^ after shaping – resulting in the formation of macropores – this is more difficult in the case of MOFs due to the low thermal stability. Janiak *et al.* employed several hydrophilic, organic binders to shape commonly used hydrostable MOFs, incorporating a freeze-drying step post the extrusion process.^203^ Küsgens *et al.* prepared HKUST-1 monoliths by extruding a slurry mixture comprising the MOF, methyl hydroxypropyl cellulose, and methoxy-functionalized siloxane ether.^204^ This method exhibited a high MOF loading per monolith, presenting an almost pure structure, compared to a coated substrate monolith. They demonstrated enhanced structural robustness, withstanding forces of up to 320 N, surpassing cordierite monoliths deposited through *in situ* growth of HKUST-1. In another study, Tsalaporta *et al.* shaped four different MOFs – UiO-66, ZIF-67, HKUST-1, and ZIF-8 – into granules using methylcellulose and bentonite as binders.^155^ ZIF-8 remained stable after the granulation process, whereas HKUST-1 and UiO-66 exhibited a reversible partial loss of crystalline morphology when pelletized with water, while ZIF-67's crystal structure was irreversibly lost.

Khabzina *et al.* produced UiO-66-COOH using a piston extruder, achieving an 89% yield and a space-time yield of 350 kg per day per m^3^ in an aqueous batch reactor without using toxic chemicals or organic solvents.^205^ They tested both freeze-granulation and extrusion for NH_3_ capture at 600–1200 ppm and relative humidity levels of 0%, 40%, and 70%. UiO-66-COOH pellets and extrudates achieved NH_3_ uptakes of 55 and 53 mg g^−1^, respectively, compared to 30 mg g^−1^ for Norit and 39 mg g^−1^ for 3 M commercial, activated carbons. The original MOF had a BET area of 710 m^2^ g^−1^, which reduced by ∼50% after mechanical compression, with pellets and extrudates showing 359 and 418 m^2^ g^−1^, respectively. Bulk densities varied, with compressed powder at 0.62 cm^3^ g^−1^, extrudates at 1.04 cm^3^ g^−1^, and beads at 0.12 cm^3^ g^−1^. Attrition tests showed less than 2% weight loss, and NH_3_ uptake remained stable after 7 days of aging at 80% humidity (34 mg cm^−3^*vs.* 33 mg cm^−3^). Regeneration at 150 °C retained 70–77% NH_3_ capacity.

Hong *et al.* used a single-screw extruder with bentonite clay as a binder to create Cr-based MIL-101 monoliths for CO_2_ adsorption.^206^ Monoliths were formed by mixing MIL-101(Cr) powder, bentonite clay, and water into a paste, which was extruded, dried first at 10 °C and then at 150 °C for 33 hours. The monoliths contained up to 75% (w/w) MIL-101 (Cr), were cut into 7 cm lengths, and had uniform channel sizes of 0.90 mm for consistent gas flow during dynamic adsorption tests. Characterization included PXRD to confirm crystal structure and SEM to reveal the cubical structure. Mercury intrusion porosimetry (MIP) indicated porosity values of 4.42% for the purified powder and 17.93% for the monoliths. Radial compression strength tests showed that monoliths with 60% and 75% weight MOF/binder ratios had elastic moduli of 10.60 N mm^−2^ and 4.97 N mm^−2^, respectively. The MIL-101(Cr) extrudates exhibited a small reduction in BET area, with 183 m^2^ g^−1^ compared to 202 m^2^ g^−1^ for the powder. However, when comparing the CO_2_ adsorption capacities at 2 bar and 25 °C, it showed a more important decrease from the 1.44 mmol g^−1^ of the powder down to 0.91 mmol g^−1^ for the extrudates. In a follow-up study, Hong *et al.* compared the performance of honeycomb MIL-101(Cr) extrudates with zeolite 13X.^110^ They found that MIL-101(Cr) monoliths have 1.3 times higher porosity than 13X zeolite monoliths. Specifically, MIL-101(Cr) monoliths demonstrated better CO_2_ mass transfer, with breakthrough and equilibrium times reduced by approximately 20% and 35%, respectively, compared to 13X zeolite monoliths. At breakthrough, the CO_2_ adsorption capacity of MIL-101(Cr) monoliths was about 37% higher (in mmol g^−1^) than that of 13X zeolite monoliths, while at equilibrium, it was about 7% lower. Overall, MIL-101(Cr) monoliths showed 1.5 times greater efficiency for CO_2_ adsorption than 13X zeolite monoliths. The study also found that higher regeneration temperatures enhanced CO_2_ adsorption capacity for both types of adsorbents.

#### 4.2.3. Spheronization

Spheronization, typically used after extrusion, is a widely employed method for producing spherical bodies. In this process, extruded structures are uniformly cut and transformed into spherical shapes through plastic deformation using a spheronizer.^207^ A typical spheronization apparatus comprises a hollow vertical cylinder and a horizontally rotating grooved ‘friction’ plate located inside. The cylindrical extrudates are dumped onto the spinning friction plate and are processed to form agglomerate shapes with nearly uniform diameters.^208^ Spherical structures typically manifest through one of two primary mechanisms. The first mechanism involves the gradual transformation of cylindrical structures, which initially exhibit sharp edges, into cylindrical shapes with rounded edges. Then, these structures evolve into dumbbell-like and elliptical particles before ultimately assuming spherical configurations. The second mechanism suggests that cylindrical structures experience torsional forces, leading to the creation of cylinders with rounded edges, which then fragment into discrete segments. Under the influence of rotational and frictional forces, these individual segments transform to form spherical particles. Importantly, this process may yield spherical particles with distinctive surface features, such as characteristic grooves or cavities.^208^ The quality of the resultant spheres and their corresponding particle size distributions are influenced by several process parameters. Notably, factors such as load, duration of the process, and rotational speed of the spheronizer play pivotal roles. For instance, as the spheronizer speed increases and the load decreases, there is a reduction in yield. Conversely, increasing both the spheronization time and load increase the yield. Furthermore, particle sizes exhibit an inverse relationship with the rotational speed. The speed parameter exerts additional influence on critical attributes such as pellet hardness, porosity, and bulk (particle) density, thereby contributing to the overall quality of the pellets.^207,208^

For example, Dhainaut *et al.* employed extrusion-spheronization to shape two MOFs, UiO-66 and UiO-66-NH_2_.^209^ They used biosourced chitosan and hydroxyethyl cellulose (HEC) as binders. They noted a preservation of the physicochemical properties of the initial powdered materials, with the BET area experiencing a reduction ranging from 5 to 33%, depending on the MOF and binder employed. Interestingly, there was a non-linear decline in the BET area, ranging from 5% to 38% reduction in BET area for binder quantities ranging from 2.0% to 5.6% by weight. Importantly, the shaping process substantially enhanced the mechanical strength of the MOFs investigated, while preserving their efficacy in capturing iodine, krypton, and xenon.^209^ In a separate study, Ren *et al.* developed a method to shape UiO-66 powder into spherical pellets ranging from 0.5 to 15 mm in diameter, using 10 wt% sucrose as a binder, rather than relying on mechanical pressing. This process, which produced kilogram-scale batches in just 30 minutes through centrifugal granulation, showed promising results.^120^ Durability tests showed no breakage after 70 consecutive drops from 0.5 m and only 5% breakage after 60 minutes of tumbling at 25 rpm. SEM images confirmed that 0.5–2 μm UiO-66 crystals were tightly bound by sucrose, maintaining interparticle space, which facilitated hydrogen diffusion, with BET areas of 674 and 1367 m^2^ g^−1^ for the spheres and powder, respectively. This decrease in porosity also reduced the hydrogen storage at 77 K and 1 bar from 1.54 wt%, powder, down to 0.85 wt% for the pellets.

#### 4.2.4. 3D-printing

**Fig. 19:**
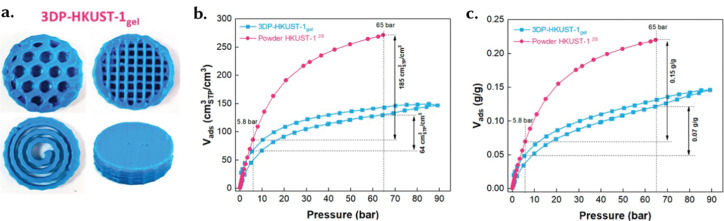
3D-printed HKUST-1 monoliths boasts impressive BET surface areas of 1134 m^2^ g^−1^ – with a substantial mesopore volume. (a) Optical images of different HKUST-1 3D printed structures. High-pressure methane adsorption tests at 90 bar and room temperature unveiled the exception capacity of the monolith. (b) Gravimetric and (c) volumetric absolute methane uptake isotherms for 3D-printed monolith and the powder forms. Adapted from ref. 212 Copyright 2019 American Chemical Society.

3D printing – also referred to as additive manufacturing – is a manufacturing process that creates 3D objects from digital files. This technique operates on the principle of layer-by-layer additive construction, where materials are incrementally deposited and built upon each other to create the final object. Numerous 3D printing methods exist, including stereolithography, digital light processing, selective laser sintering, and binder jetting, among others.^210^ This precision fabrication technology offers multiple advantages, such as unparalleled design flexibility, rapid production capabilities, and the ability to generate sophisticated geometries that would be exceedingly challenging to achieve with traditional manufacturing methods. Notably, 3D-printing has found extensive applications in the biomedical field. For example, Hsieh *et al.* reported how polyurethane–gelatin hydrogels’ mechanical properties augmented when adding ZIF-8 as a network enhancer.^211^ The addition of a small quantity of MOF (≤750 mg mL^−1^) notably enhanced modulus features, shear-thinning behavior, and structural stability without compromising printing properties or water capacity. For the 3D printing, various amounts of 500–900 nm ZIF-8 crystals (50, 125, 1250, and 3750 mg mL^−1^) were incorporated into a polyurethane/gelatin bio-ink to improve modulus. An optimal MOF concentration of 875 mg mL^−1^ was determined for cell survival and proliferation. The resulting 3D-printed MOF composite bio-inks demonstrated excellent stackability and printability for constructing blood vessels and ear-shaped structures. Lim *et al.* used colloidal gels made of ethanol and HKUST-1 NPs as inks to craft 3D printed MOF monoliths (Fig. 19a).^212^ The 3D-printed HKUST-1 structure showed a BET area of 1134 m^2^ g^−1^ along with a 0.61 cm^3^ g^−1^ total pore volume. (Fig. 19b and c). High-pressure CH_4_ adsorption at 65 bar and room temperature showed 271 and 131 cm^3^(STP) cm^−3^ for the 3D-printed HKUST-1 structure and powder, respectively (Fig. 19c). The former adsorption capacity is similar to the reported values of free standing, sol–gel HKUST-1 monoliths.^18^ The printed monolith showed a reasonable hardness of 42 Vickers Hardness (HV). Moreover, the structural integrity was deemed suitable for gas storage applications, offering promising avenues for various configurations including microreactors and adsorbent beds.

#### 4.2.5. Phase inversion and hydrogelation

Phase inversion is a process for the transformation of a thermodynamically stable polymer solution from a fluidic state to a solidified state. This transformation is initiated by the liquid–liquid de-mixing phenomenon, where the initially homogeneous polymer solution segregates into two distinct phases. One phase becomes enriched with polymer molecules, forming a dense and concentrated region, while the other phase exhibits a lower concentration of polymers.^213^ As this segregation process progresses, the polymer-rich phase undergoes solidification through various mechanisms, such as gelation or crystallization. These processes lead to the formation of a sturdy and continuous solid membrane structure within the material. Concurrently, the phase containing a lower concentration of polymers facilitates the creation of pores or voids within the material matrix. These pores contribute to the overall porosity and permeability of the resulting membrane. The interplay between the polymer-rich and polymer-lean phases during phase inversion ultimately yields materials with tailored morphological features optimized for applications of interest in the present context.^213^ The de-mixing process can be induced by four common techniques: reducing the temperature, immersing the material in nonsolvent baths, eliminating volatile solvents from the solution, or precipitating from a vapor-phase.^213,214^ Among these, one prevalent method involves immersion precipitation, where the polymer solution is immersed in nonsolvent baths. Nonsolvent baths are solutions that do not dissolve the polymer but instead induce de-mixing. These baths typically consist of substances in which the polymer has low solubility or does not dissolve at all. For example, water can often act as a nonsolvent for many organic polymers. When the polymer solution comes into contact with the nonsolvent bath, it triggers de-mixing by altering the solvent-polymer-nonsolvent interactions. This change in interactions leads to the formation of two distinct phases: one rich in the polymer and the other containing a lower concentration of polymer. The bath provides an environment conducive to the controlled separation of these phases. By carefully selecting the composition and conditions of the nonsolvent bath, it becomes possible to exercise control over the de-mixing process, allowing for the fabrication of membranes with desired morphological characteristics.^213^ While there are several thermodynamic considerations based on ternary phase diagrams of polymer/solvent/nonsolvent systems and kinetic factors related to mass transfer rates of solvent and nonsolvent, these concepts fall beyond the scope of this review. Readers interested in expanding on this topic are encouraged to refer to an excellent review by Holda and Vankelecom.^213^

Phase inversion has been used for the shaping of MOFs to exploit their catalytic properties for the neutralization of chemical warfare agents (CWAs). Peterson *et al.*^215^ developed reactive, MOF–polymer composite beads, with a size ranging from 300 μm to 2 mm, using phase inverted poly(styrene-*block*-ethylene-ran-butylene-*block*-styrene) (SEBS). Due to the bulky nature of the polystyrene (PS) blocks, there was a low infiltration of the polymer into the pores of the MOF, thereby preserving the core functionality while imparting viscoelasticity to the resulting composite material. The resulting composite showed better CWA removal capabilities and reactivity in comparison to activated carbon fabrics – which were the previous state-of-the-art. Along similar lines, Stylaniou *et al.*^216^ developed spherical composite beads (MOF@polymer beads) from UiO-66-NH_2_, UiO-66-pyridine, and UiO-67-(NH_2_)_2_ combined with poly(vinylidenefluoride) (PVDF), PS, and poly(ether sulfone) (PES).^217^ Here, the MOF was first synthesized and then combined with the polymer. By doing so, it retains the crystallinity of the MOF and the accessibility to the pores. Composite beads formed from UiO-66-NH_2_ and PES showed good catalytic performance in converting dimethyl *p*-nitrophenylphosphate (DMNP) to dimethyl phosphate (DMP), achieving a conversion rate of 62% in just 5 minutes. This catalytic efficiency may be attributed to the presence of interconnected macropores. Additionally, the composite beads's activity was kept over three cycles.

**Fig. 20:**
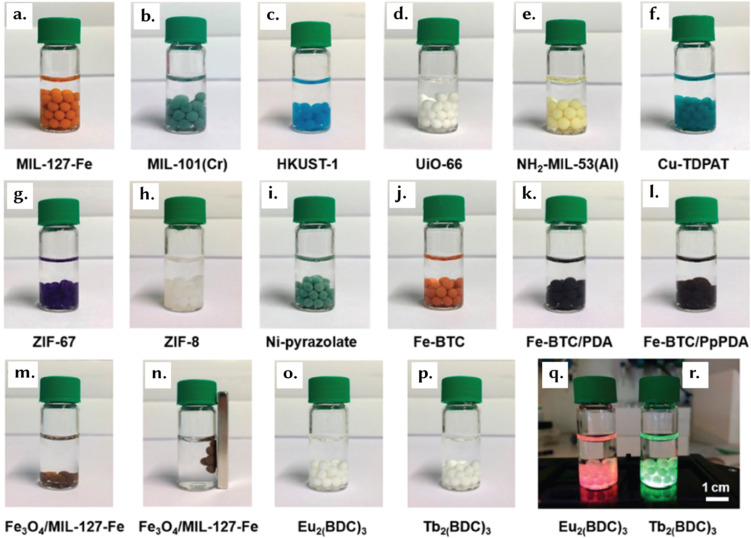
A polymerization process using biocompatible and biodegradable monomers, cross-linked with calcium ions has been developed for the preparation of MOF–polymer composite beads. Optical images of beads of (a) MIL-127-Fe, (b) MIL-101(Cr), (c) HKUST-1, (d) UiO-66, (e) NH_2_-MIL-53(Al), (f) CuTDPAT, (g) ZIF-67, (h) ZIF-8, (i) Ni-pyrazolate, (j) Fe-BTC, (k) Fe-BTC/PDA, (l) Fe-BTC/PpPDA, (m) and (n) magnetic Fe_3_O_4_/MIL127-Fe, (o) Eu_2_(BDC)_3_, and (p) Tb_2_(BDC)_3_ as well as (q) Eu_2_(BDC)_3_ and (r) Tb_2_(BDC)_3_ under ultraviolet illumination with a wavelength of 254 nm. Adapted with permission from ref. 219 Copyright 2022, American Chemical Society.

Beyond the phase-inversion method, there have been several attempts to shape MOFs as composite beads, such as using gelation-based techniques. For example, Valizadeh *et al.* synthesized a UiO-66 analogue using double amino functional groups in the linker (UiO-66(NH_2_)_2_) and then shaped it into MOF@PES beads for the removal of Cr(vi) from water.^218^ The formation of composite beads addresses several challenges such as clogging, pressure drop, and material loss, which are often encountered when loading the powder into columns. The composite recorded a high Cr(vi) uptake of 135 mg g^−1^, while being fully recyclable – tested in real-world samples. Additionally, the integrated process, which was performed in a glass column equipped with a visible light source, allowed for the photoreduction of Cr(vi) solution to less toxic Cr(iii) species during adsorbent regeneration, an interesting approach for Cr(vi) removal in a single continuous process.^218^ Yang *et al.*^219^ developed a polymerization strategy for the preparation of MOF–polymer composite beads using biocompatible and biodegradable poly(acrylic acid) (PAA) and sodium alginate monomers. The method involved the formation of double-cross-linked networks of PAA, sodium alginate and Ca^+2^ ions in water. The introduction of PAA made the beads highly stable due to hydrogen-bonding and ionic interactions – making them a promising strategy for liquid separations. The strategy allowed the formation of stable composite beads for 15 structurally diverse MOF systems (Fig. 20), MIL-101(Cr), MIL-100(Fe), HKUST-1, UiO-66, ZIF-8, ZIF-67, and MIL-100(Fe)/PDA; the beads exhibited a Pd uptake of 498 mg g^−1^. The method was applicable for large-scale structuring of the reticular porous materials using a continuous flow system driven by a peristaltic pump.^219^ An alternative solution involves the utilization of MOF–cellulose composite beads.^220^ By embedding MOFs within biodegradable sodium CMC, MOF–cellulose composite beads were created. The drying was done *via* two different methods of heat drying and freeze-drying. This structure maintains MOF crystallinity and porosity (with the drop in the BET area being less than 20% for both drying techniques compared with the original powder) while providing the composite beads with robust mechanical properties. To demonstrate practicality, they showed that MIL-100/CMC-HD composite beads effectively degrade more than 95% of dyes and are amenable to multiple cycles of reuse.

#### 4.2.6. Glass formation

While MOFs have long captivated researchers with their porosity and crystalline structure, there has been a growing interest in exploring their amorphized phases, revealing new dimensions of versatility and potential applications. This area of research was initiated by pioneering work from Bennett, Goodwin and Cheetham, who first demonstrated the amorphization of ZIF-4, a prominent MOF variant.^221^ Their study laid the foundation for deeper investigations into the behavior and properties of amorphized MOFs. Over the years, Bennett, Horike and others have expanded this work, systematically growing upon the early findings and coining the term ‘MOF glasses.’ Glass formation in materials like coordination polymers (CPs) and MOFs can be achieved through various methods beyond traditional melt-cooling processes. Techniques such as melt-quenching, mechanical induction (namely *via* ball-milling), and direct glass synthesis have been successfully used to create consistent amorphous structures. Each of these techniques will be briefly introduced here. In a melt-quenching process, a crystalline MOF is heated to temperatures above its melting point in order to obtain a ‘melt’ state. The melting temperatures of MOFs can vary based on their specific composition and structure. Typically, MOFs exhibit relatively low melting points compared to conventional inorganic materials. Generally, the melting point of MOFs falls within the range of 350–750 °C. This melt state is then cooled rapidly – in a process called ‘vitrification’ – in order to obtain a ‘glassy’ state. This is only achievable when the melt state is stable, which in itself is not a usual phenomenon.^142^ Indeed, this phenomenon has been just observed for a few classes of families of CPs/MOF, including some phosphate–azole frameworks, ZIFs, thiocyanate and nitrile-based frameworks, and metal-bis(acetamide) frameworks. Alternately, a glassy state may be induced *via* the introduction of mechanical stimuli through processes such as ball-milling. Furthermore, it is possible to directly synthesize amorphous MOFs exhibiting glassy behavior using methods analogous to the sol–gel method discussed in Section 4.1.1.^142^ For more detailed discussions on these aspects, we refer the reader to excellent reviews dedicated to glassy MOFs and the methods for their preparation.^34,142^

In terms of the performance and application of MOF glasses, Wang *et al.* developed a glass-based membrane from the mixed-linker framework ZIF-62.^147^ The framework was chosen for its glass-forming ability, as it undergoes a melting process prior to decomposition without interfering with its immediate recrystallization. While the ZIF-62 glass retained some porosity, there was a noticeable drop in the gas uptakes from 18.5 to 11 cm^3^ g^−1^ for the crystalline and the glass, respectively, for CO_2_ at 1 bar and 293 K; 10 and 2.6 cm^3^ g^−1^ for the crystalline and the glass, respectively, for CH_4_ at 1 bar and 293 K; 2.4 and 0.7 cm^3^ g^−1^ for the crystalline and the glass, respectively, for N_2_ at 1 bar and 293 K. The permeance rates at room temperature for H_2_, CO_2_, N_2_, and CH_4_ were of 22, 9.7, 0.41, and 0.37 × 10^−9^ mol m^−2^ s^−1^ Pa^−1^, respectively. They additionally fabricated a composite MOF glass membrane on porous ceramic alumina support using a melt-quenching approach. They, however, faced challenges with regards to an even spread of the melt on the support due to its high viscosity. Nonetheless, the resulting membranes were grain-boundary free, having the potential for long-term stability – they demonstrated no loss in permeance and selectivity for over two days. They exhibited excellent separation performance for H_2_/CH_4_, CO_2_/N_2_ and CO_2_/CH_4_, with selectivities of 50.7, 34.5, and 36.6, respectively.^147^ With regards to the somewhat rarer *in situ* glass formation, Yaghi and co-workers showed how a slow evaporation of a solution containing Ti-oxo clusters, fumaric acid, and *m*-cresol in a mixture of ethanol and tetrahydrofuran led to the formation of carboxylate linkages between the cluster and the linker, resulting in a transparent glass.^222^ The glass – named Ti-Fum – had a record-high BET area of 923 m^2^ g^−1^, a value much higher than typical MOF glasses.^222^

## 5. Considerations for industrial translation

### 5.1. Influence of structuring methods on structural integrity under extreme conditions

The industrial implementation of porous reticular materials requires not only a high degree of control over their structuring but also a thorough understanding of how these methods influence their mechanical robustness, chemical stability, and long-term durability under extreme operational conditions. As discussed in previous sections, in industrial applications, these materials are often exposed to harsh environments that can lead to framework degradation, pore blockage or collapse, and loss of crystallinity.^33^ Mechanical stability is particularly important for gas storage, separation, and catalysis, where materials experience high pressures during adsorption and desorption cycles. Structuring methods such as densification, extrusion, and sol–gel monolith formation may significantly impact the ability of these materials to withstand mechanical stress. Densification techniques are commonly used to improve volumetric performance but can result in reduced porosity and pore connectivity.^99^ Recent studies have shown that hierarchical structuring, such as templated assembly and freeze-casting, can enhance mechanical stability by introducing reinforcing architectures that prevent pore collapse under pressure while maintaining high porosity.^203,216^ Additionally, hybrid structuring approaches combining sol–gel processing have demonstrated improved resistance while retaining surface area and functionality.^96^ For instance, the sol–gel processed _mono_HKUST-1 displayed a Young's modulus which matches its conventional powder counterpart, while having a 130% hardness due to its higher density.^18^ Another strategy to mitigate pressure-induced framework collapse involves incorporating secondary support structures or polymer binders into monolithic frameworks. Studies on polymer/MOF composites have reported improved mechanical stability while maintaining adsorption performance, particularly in gas storage applications.^219,223^ However, the main challenge here lies in optimizing binder selection to avoid pore blockage and loss of accessible surface area.

Conventional MOFs with weak metal–ligand bonds, such as Zn-based frameworks with carboxylic acid based linkers, start degrading above 300 °C, while higher-temperature-resistant MOFs like UiO-66 offer greater stability due to their stronger coordination bonds.^224,225^ However, for long-term industrial applications such as catalysis, membrane separation, and gas capture, MOFs should not operate above 150 °C to prevent structural degradation. The structuring method plays a crucial role in thermal resilience, as shaping techniques can either reinforce or compromise mechanical integrity. COFs, which rely on covalent rather than coordination bonds, generally exhibit higher thermal stability than MOFs.^9^ However, structured COF forms, including fibers, membranes, and aerogels, may become unstable under extreme heat and pressure.^226^ Strategies such as cross-linking and carbonization have been explored to enhance their durability while maintaining porosity. Pyrolyzed COF monoliths have shown excellent thermal stability in catalytic applications, making them promising for high-temperature environments.^227,228^ To ensure industrial viability, careful selection of structuring techniques and thermal management strategies is essential for maintaining stability and functionality over extended periods.

Industrial applications such as carbon capture and water harvesting require reticular materials to function under humid/aqueous environments. However, exposure to moisture and acidic conditions often leads to hydrolysis and structural degradation in most MOFs.^229^ Post-synthetic modifications, such as fluorination or hydrophobic coatings, have been shown to enhance the water stability of MOFs while maintaining their adsorption properties.^230^ The structuring process may also influence hydrothermal stability; for example, blending MOFs into a polymer matrix may improve resistance to water-induced degradation compared to pristine MOF powders due to the protective polymer layer.^96^ In contrast, COFs generally exhibit greater water stability due to their robust covalent linkages, yet their structured forms, such as membranes, can still be prone to swelling and pore blockage under prolonged water exposure. Advances in cross-linked COF membranes have addressed some of these issues, enhancing their thermal stability, mechanical stability and anti-swelling properties.^231^ Furthermore, hybridization with hydrophobic fillers such as graphene has been explored to improve moisture tolerance in structured COFs.^232^

Despite the progress in improving the robustness of structured reticular materials, several unresolved challenges remain, and as such, future research should focus on: (i) developing advanced structuring techniques that preserve porosity while enhancing framework stability under extreme conditions; methods using standard unit operations will ensure low capex; (ii) investigating long-term performance through accelerated aging studies that simulate real-world industrial conditions; understanding the degradation pathways of structured materials will enable the design of more resilient frameworks;^233^ and (iii) establishing standardized testing protocols for evaluating the stability of structured reticular materials, which will facilitate the direct comparison of structuring techniques and accelerate their transition to commercial use.

### 5.2. Structured hybrid/composite materials

The integration of structured composite materials – where reticular frameworks are combined with polymers, metal oxides, or carbon-based supports – has been receiving attention as a means to enhance durability, scalability, and functional performance. For instance, MOF–polymer composites are being investigated due to their ability to retain the porosity and selectivity of MOFs while improving mechanical robustness and ease of processing.^234^ Polymer-supported MOF membranes and beads have demonstrated enhanced durability for gas separation, water purification, and catalysis.^234^ Studies have shown that polymer-grafted MOFs exhibit superior flexibility and adhesion properties, making them ideal candidates for coatings and sensor applications. Similarly, MOF–polymer aerogels have been developed for high-performance adsorption and catalysis due to their lightweight structure and large accessible surface area.^134^ The incorporation of metal and metal-oxide nanoparticles into MOFs has led to the realization of core–shell hybrid structures, enhancing catalytic and sensing properties. For instance, the encapsulation of Pd, Pt, and Au nanoparticles within MOFs has resulted in improved catalytic efficiency for hydrogenation and oxidation reactions.^235^ Meanwhile, SnO_2_@MOF hybrids have exhibited significant performance in gas sensing due to the synergistic interactions between the porous architecture of MOFs and the semiconductor properties of SnO_2_.^236^ Additionally, magnetic MOF hybrids have been explored for their applications in environmental remediation, where their magnetic properties allow for easy separation and recovery after use.^237^

A key challenge in industrial applications of porous materials is their handling in large-scale reactors. MOF–alumina composites have demonstrated enhanced mechanical strength, making them suitable for fixed-bed adsorption systems.^238^ Similarly, carbon-based MOF films, such as UTSA-16/carbon hybrids, offer improved CO_2_ adsorption capacity and faster adsorption kinetics – expanding their applicability in gas storage and separation.^239^ Developments in spray-dried MOF–polymer hybrids have enabled the formation of spherical granules with uniform porosity, facilitating their direct use in adsorption and catalysis.

A key issue in MOF composites is the optimization of interfacial interactions between MOFs and their composite phases to prevent phase separation and degradation. Fabrication techniques, such as ALD and *in situ* polymerization, are being explored to achieve better compatibility and stability. Looking ahead, the integration of machine learning and computational modeling in the design of structured composite materials will be important for optimizing processing conditions and predicting material stability. Furthermore, the adoption of sustainable and solvent-free processing techniques will help in aligning MOF-based composites with green manufacturing principles, promoting their broader adoption across industrial sectors.

### 5.3. Defects and their potential industrial implications

Structural defects may arise during shaping and processing and consequently may significantly influence their industrial performance. For example, mechanical compression, extrusion, solvent evaporation, additive manufacturing, and thermal processing introduce grain boundaries, fractures, voids, and partial amorphization – as discussed in previous sections – which can alter porosity, adsorption efficiency, and mechanical stability. While defects can compromise functionality, controlled defect engineering has, at times, been used as a tool to enhance performance.^240^ For instance, structural defects in UiO-66-NH_2_ xerogels enhance CO_2_ adsorption and separation kinetics, while controlled linker vacancies in catalytic MOFs boost efficiency.^241,242^ In conductive MOFs, defect pathways can facilitate electron mobility, while targeted defect introduction improves hydrothermal stability, preventing framework degradation in harsh environments.^120,243^ Scalable defect control, however, remains a challenge, requiring predictive computational models such as density functional theory (DFT) and machine learning for rational defect design. Long-term stability of defect-engineered materials must again be evaluated through accelerated aging studies and real-time process monitoring.

### 5.4. Cost reduction and sustainable structuring

For structured reticular materials to be widely adopted in industry, shaping techniques must be cost-effective, scalable, and compatible with existing manufacturing processes. Traditional methods like extrusion, pelletization, and granulation remain the most practical, offering high throughput and continuous processing with well-controlled mechanical properties. Compared to spray drying, which has high energy demands and material losses, extrusion is a more economical and scalable solution for producing mechanically stable structures. Incorporating biopolymer-based binders like alginate or cellulose provides an eco-friendly alternative to synthetic additives, enhancing sustainability and mechanical performance while maintaining porosity as high as possible. Granulation and pelletization ensure high-scale production with sufficient mechanical strength for packed-bed applications. Sol–gel synthesis also offers advantages, particularly in producing monoliths with high mechanical resistance and controlled porosity. These monoliths can be molded into various shapes while maintaining structural integrity, making them useful for adsorption, catalysis, and separation applications. Their high density and mechanical stability allow direct use in gas storage and separation without additional shaping steps. However, optimizing sol–gel processes for large-scale production remains a challenge due to potential shrinkage and cracking. While 3D printing allows for complex geometries with tunable porosity, it currently remains impractical for industrial-scale production due to high capital expenditures, slow processing rates, and expensive feedstock. Unlike extrusion, which supports bulk material processing, 3D printing operates in batch mode, making it unsuitable for high-volume applications. Though beneficial for prototyping, the economic and technical barriers to scaling additive manufacturing outweigh its advantages for mainstream industrial use. Mechanochemical synthesis and shaping has been explored as a solvent-free alternative, but it struggles to match the structural integrity and porosity control achieved by wet chemistry using green solvents and is not a universal method for every material. While liquid-free processing reduces waste and improves sustainability, optimizing wet-chemistry-based shaping with environmentally friendly solvents remains – probably – the more reliable and scalable approach. Industrial adoption also depends on addressing regulatory concerns, including compliance with environmental regulations, toxicity assessments, and stability requirements. Integrating lifecycle assessments, solvent recovery, and sustainable waste management into production workflows will ensure alignment with evolving regulations.

## 6. Outlook

**Fig. 21:**
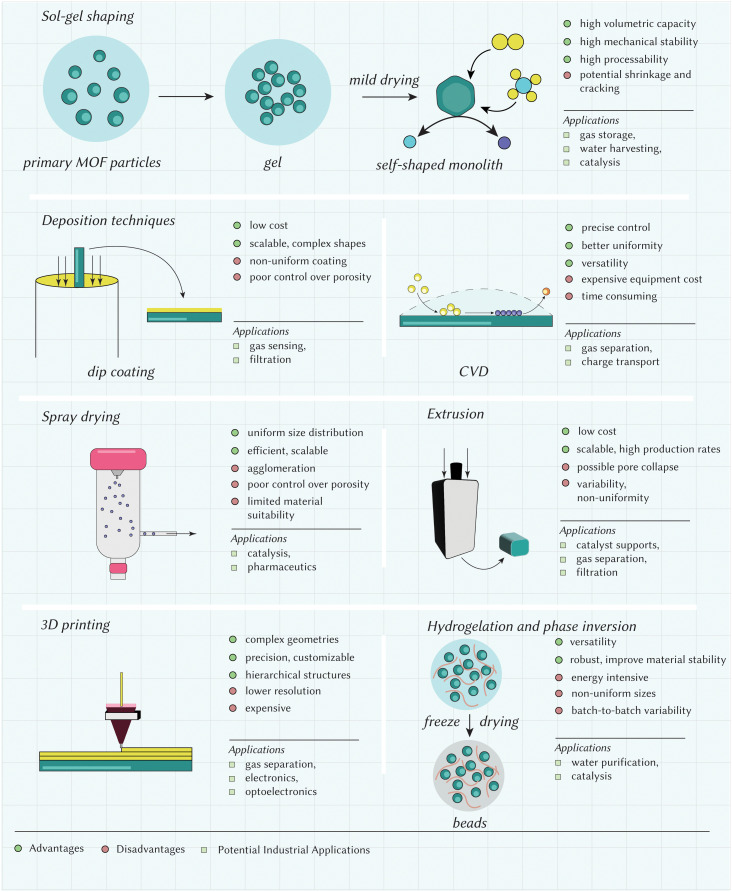
A ‘blueprint’ summarising the key advantages and disadvantages, along with potential industrial applications of the main structuring techniques discussed in the review.

Decades of research have positioned porous reticular materials at the forefront of adsorption-based energy applications, showing promising performance for handling challenges posed by new gas storage, separation, catalysis, and sensing-based applications. Our understanding of these materials – especially MOFs and COFs – have matured to the stage where we have the capability of establishing a high-degree of control over the structure, composition, functionality, and porosity of these materials for the application under consideration. Despite excellent performance metrics and promise, the use of reticular materials is – with some exceptions – largely limited to academic pursuits. While there are several components responsible for this translational gap, we have identified here the structuring and densification of these materials as a critical bottleneck. We have discussed progress in the structuring of porous reticular materials and have provided comprehensive insights into tackling the challenges posed by the poor structuring of these materials. We have highlighted principles governing the hierarchical synthesis of these materials at the microscale, emphasizing the importance of establishing strong control over the quality of the material. In turn, we have presented the landscape of shapes and techniques available for structuring porous reticular materials at the macroscale, highlighting their unique advantages and disadvantages (Fig. 21).

Among the different shaping techniques, sol–gel synthesis stands out as a versatile method offering precise control over pore size and structure. In gas storage applications, the resulting high densities of sol–gel-shaped materials translate to remarkable volumetric capacities, crucial for confined spaces where maximizing gas storage within a limited volume is essential. Of course, this not only applies to gas storage but also to gas separation, where minimising the footprint is critical to reduce capital and operation costs. Having said that, there are certain challenges that exist enroute to industrial translation. If not done adequately, the high packing density of these materials can result in low diffusion coefficients for reactants and adsorbates, which may hinder performance in dynamic processes. Notably, in some cases, the adsorption kinetics can be higher in monoliths due to the better heat conductivity of densified bodies compared to powder.^158^ Additionally, the synthesis of large-scale sol–gel materials may encounter issues pertaining to shrinkage and cracking – limiting scalability for mass production.

Dip coating is particularly suitable for creating thin films on complex shapes. It is well-suited for applications requiring effective sensors and membranes. In gas sensing, dip coating enables the deposition of sensitive sensing materials onto sensor substrates, facilitating the selective detection of target gases. However, controlling film thickness and porosity can pose challenges, potentially affecting sensor performance and reliability. Additionally, deposition techniques, such as PVD and CVD and their variants, offer precise control over film thickness, making them ideal for gas separation membranes. They allow for the deposition of ultra-thin films with customized properties, enabling efficient gas separation processes. However, implementing deposition techniques often requires specialized equipment and can be complex and costly.

Careful optimization of spray drying allows producing porous particles with a uniform size distribution. However, challenges arise in controlling pore structure and preventing agglomeration, which can impact material performance. Agglomeration of particles during the drying process can lead to uneven distribution of pores and reduce the overall surface area, limiting the effectiveness of the material in gas storage applications. On the other hand, extrusion is cost-effective and suitable for large-scale production of porous reticular materials, particularly for applications requiring catalyst supports and gas separation adsorbents. However, compared to other structuring methods, these techniques may result in reduced porosity and surface area as well as gas diffusivity due to compaction during mechanical compression. Despite these drawbacks, their simplicity and cost-effectiveness make them attractive options for industrial-scale production of materials.

3D printing stands out for its ability to fabricate complex geometries and tailor pore structures with precision, making it highly advantageous for various applications. In gas sensing, 3D printing allows for the creation of custom sensor designs with specific sensitivity and selectivity. By controlling the layout and composition of sensor components, it enables the development of sensors optimized for detecting target gases in specific environments. However, the adoption of 3D printing in gas sensing may be hindered by the need for specialized equipment and materials, as well as potential limitations in resolution compared to conventional bottom-up approaches as well as limitations in scalability. If cost is justified, the flexibility and customization offered by 3D printing make it a promising technique for advancing gas sensing technology. Phase inversion and hydrogelation techniques provide versatile approaches for producing porous structures, encompassing both membranes and beads tailored for adsorbent and catalyst applications. These methods enable the controlled phase separation of polymer solutions or hydrogels, facilitating the creation of membranes with precisely controlled pore sizes and distributions. Widely utilized in membrane fabrication, these membranes exhibit tuneable characteristics ideal for efficient gas separation based on differences in molecular size and affinity. However, issues such as batch-to-batch variability, non-uniform bead sizes, and difficulties in achieving consistent pore structures pose obstacles to widespread industrial adoption. Despite these challenges, ongoing research efforts aim to address these limitations and enhance the scalability and reliability of phase inversion and hydrogelation techniques for industrial-scale production. These efforts include exploring novel methods for process control, advanced characterization techniques, and the development of more robust materials to overcome the current limitations and accelerate the industrial adoption of these promising techniques.

A key avenue for overcoming these challenges lies in computational modeling and simulations, which provide predictive insights into structuring methodologies. Traditional experimental approaches to structuring reticular materials often rely on iterative trial-and-error processes, which are time-consuming and inefficient. The integration of density functional theory (DFT), molecular dynamics (MD), and finite element modeling (FEM) has allowed researchers to simulate the effects of mechanical compression, extrusion, and thermal processing on porosity, mechanical stability, and adsorption behavior. Computational fluid dynamics (CFD) simulations further enable the study of mass transfer and diffusion properties within structured monoliths, membranes, and aerogels—critical for optimizing gas separation and catalytic applications. Beyond predicting processing outcomes, computational approaches also aid in defect engineering in shaped materials. While structuring techniques can introduce defects such as missing linkers, grain boundaries, or pore collapse, simulations provide a means to predict, control, and even leverage these defects for enhanced functionality. Furthermore, machine learning algorithms trained on large experimental datasets can optimize shaping parameters such as pressure, binder content, and solvent evaporation rates, enabling faster and more efficient material development.

Despite the progress made in structuring porous reticular materials, several key challenges must be addressed before their large-scale industrial adoption becomes feasible. One of the most pressing concerns is the ability to maintain structural integrity and porosity throughout shaping and densification. Many conventional shaping techniques compromise essential properties such as surface area, pore connectivity, and mechanical stability. Although recent advancements in additive manufacturing, templated synthesis, and sol–gel methods have demonstrated promise, further optimization is required to ensure these techniques preserve functional properties across both micro- and macroscales. Future research should focus on developing shaping strategies that strike a balance between densification and porosity retention, minimizing pore collapse while maintaining sufficient mechanical strength and stability for industrial applications.

Another major challenge lies in the development of scalable and cost-effective structuring methods. Advanced techniques such as 3D printing and chemical vapor deposition offer precise control over material structuring, but their reliance on expensive precursors and specialized equipment limits their feasibility for large-scale production. Furthermore, understanding the impact of processing parameters – such as pressure, temperature, and precursor concentration – on the final properties of shaped materials will be crucial in establishing robust and reproducible structuring methodologies.

Long-term stability is another critical concern, as structured MOFs and COFs must withstand real-world operational conditions, including fluctuations in humidity, temperature, and mechanical stress. While much research has been dedicated to optimizing initial material performance, less is known about how these materials degrade over time in industrial harsh environments. Degradation mechanisms such as framework collapse, chemical instability, and fouling must be systematically investigated. Future efforts should prioritize accelerated aging studies, *in situ* characterization techniques, and the development of protective coatings to enhance material durability, ensuring their reliability for practical applications.

In addition to experimental advancements, improved theoretical and computational models are essential for guiding the structuring of porous materials. Existing models often focus on idealized structures, whereas real-world applications involve complex morphologies and heterogeneous environments. The integration of machine learning and computational simulations could provide predictive insights into key parameters such as pore connectivity, mechanical resilience, and diffusion efficiency, allowing for the rational design of structured MOFs and COFs. By coupling experimental research with computational tools, researchers can accelerate the development of highly functional, structured reticular materials tailored for specific industrial needs.

Finally, sustainability and recyclability must be at the forefront of future research in structured reticular materials. As industries transition towards circular economy principles, it is critical to develop environmentally friendly synthesis routes that minimize solvent use, energy consumption, and waste generation. Additionally, research into the regeneration, reuse, and recyclability of shaped MOFs and COFs will be crucial for ensuring long-term sustainability. Addressing these challenges will facilitate the transition of structured porous materials from niche academic research to widespread industrial applications, ultimately playing a transformative role in energy storage, environmental remediation, and next-generation catalysis.

Looking at a broader perspective, there is a clear need for developing a deeper fundamental appreciation of how properties at the microscale influence the behaviour at the macroscale. This appreciation would serve to guide a rational, judicious selection of structuring techniques for the effective implementation of clearly promising materials at industrial scales. Here, a judicious combination of the techniques that we have discussed in the present context may also hold promise. As the development of these materials advances, it is a sincere hope that their structuring is not forgotten in the process.

## Data availability

No primary research results and no new data were generated or analysed in the context of this review article.

## Conflicts of interest

D. F.-J. has a financial interest in the start-up company Immaterial, which is seeking to commercialize metal–organic frameworks.
